# Heterogeneous suppressive effect of *Wolbachia* incompatible insect technique coupled with sterile insect technique across time and historical *Ae. aegypti* abundance - using distributional synthetic controls

**DOI:** 10.1371/journal.pcbi.1014355

**Published:** 2026-06-22

**Authors:** Yichen Zhai, Chia-Chen Chang, Zhiyong Xi, Cheong Huat Tan, Lee Ching Ng, Jue Tao Lim

**Affiliations:** 1 Lee Kong Chian School of Medicine, Nanyang Technological University, Singapore, Singapore; 2 Environmental Health Institute, National Environment Agency, Singapore, Singapore; 3 Department of Biological Sciences, National University of Singapore, Singapore, Singapore; 4 College of Natural Sciences, Michigan State University, East Lansing, Michigan, United States of America; 5 School of Biological Sciences, Nanyang Technological University, Singapore, Singapore; University of Melbourne, AUSTRALIA

## Abstract

**Background:**

Biological control tools such as *Wolbachia* incompatible-insect technique, are a promising class of interventions to modify and suppress *Aedes aegypti* mosquitoes to reduce risk of *Aedes*-borne diseases. Due to the spatial nature of the intervention, intervention effects can be spatio-temporally heterogeneous. Yet, most evaluations of field-based technologies rely on average treatment effects, which preclude characterization and understanding of treatment effect heterogeneities and the factors influencing it.

**Methods:**

Here, we developed a causal inference framework using distributional synthetic controls to explicitly account for spatio-temporal trap-level mosquito abundance data to ascertain the entomological efficacy of *Wolbachia* in suppressing *Ae. aegypti* abundance. This method is able to construct counterfactual distributions of intervened areas, provide detailed comparisons to actual distributions and quantify treatment effects of the intervention on mosquito abundance over different quantiles. By employing our framework to trap-level mosquito abundance data from 66,712 unique mosquito traps routinely maintained and measured twice a week, and a large-scale field trial of *Wolbachia* incompatible-insect technique coupled with sterile insect technique (IIT-SIT) in Singapore, we (**1**) quantified heterogeneous treatment effects for IIT-SIT across the time-since-intervention, over the traps’ historical mosquito abundance, over calendar time, (**2**) quantified whether elimination of wild-type *Aedes aegypti* was possible in intervention locations and (**3**) addressed if suppressive effects in spillover locations adjacent to directly intervened locations were heterogeneous.

**Results:**

IIT-SIT interventions led to a strong suppressive effect on adult *Aedes aegypti* abundance. From the onset of intervention in directly treated locations, sector-specific intervention effectiveness (IE) ranged from 24.04% in the earliest treatment period, and reached 86.08% in the latest treatment period. Raw reductions in *aegypti* abundance were also found to increase over time as sectors were intervened over longer time periods. In spillover sectors, IE was lower in magnitude and more variable, but average IE reached a maximum of 78.08% in 2-years post-treatment. *Wolbachia* interventions also led to an increase in the percentage of traps recording no mosquitoes from 6.8% at the start of intervention to 33.01% 124-weeks post-intervention. We found that IE was higher in sectors with lower historical mosquito abundance. However, IE converged across sectors with different historical mosquito abundance as intervention time increased.

**Conclusion:**

This study revealed spatial heterogeneities in suppressing wild-type female *Ae. aegypti* by IIT-SIT and provided strong evidence that IIT-SIT can drastically suppress wild-type *Ae. aegypti* populations despite heterogeneous treatment effects over time.

## Author summary

*Aedes*-borne diseases transmit across tropical and sub-tropical regions of the world, with the most important being dengue threatening half the global population. Vector control plays a fundamental role in reducing transmission and infection by reducing vector populations. Previous studies have demonstrated that a promising biological control technique called IIT-SIT can efficiently suppress *Ae. aegypti* population and have examined its effects across different durations of suppression. However, less attention has been given on how this suppressive effect varies spatio-temporally. We therefore developed a framework which can characterize differences in these intervention effects on a fine-spatio-temporal scale. This framework can provide new insights on the spatio-temporal heterogeneity of the treatment on mosquito abundance. We identified a general suppression pattern across different levels of mosquito abundance and showed that elimination of wild-type mosquitoes was not possible in the intervened areas. We also demonstrated similar but weaker suppression effects in locations adjacent to intervention areas. These findings illustrate the spatio-temporal heterogeneity of vector control and can provide guidance for optimizing resource allocation in future intervention implementation.

## Introduction

Dengue fever is one of the highest burden vector-borne diseases of major public health concern. The causative agent is the dengue virus and is transmitted primarily by the *Aedes aegypti (Ae. aegypti)* and *Aedes albopictus (Ae. albopictus)* vectors. Due to rapid urbanisation and climate change, the at-risk population and incidence of dengue have steadily increased in the past decades. Pharmaceutical options for prevention or treatment remain limited; the primary means to prevent *Aedes*-borne diseases, including chikungunya, dengue, yellow fever and Zika, is therefore vector-control. Incompatible Insect Technique coupled with the Sterile Insect Technique (IIT-SIT) is an emerging vector control method aimed at suppressing wild-type mosquito populations. IIT utilizes *Wolbachia*-infected male mosquitoes that, when released, produce inviable offspring upon mating with wild-type females. However, imperfect sex-sorting can lead to stable establishment of the released *Wolbachia* strain in the field due to unintentional release of fertile *Wolbachia*-infected female mosquitoes [1]. Therefore, SIT via low-dose irradiation to target female mosquito fertility is used to mitigate this risk. IIT-SIT offers several advantages over conventional vector control strategies such as insecticide-based approaches, and complements environmental management. Insecticide resistance can reduce the efficacy of chemical interventions. IIT-SIT complements traditional community-based source reduction efforts which may not reach cryptic breeding sites [2]. In addition, IIT-SIT provides a species-specific alternative with few off-target effects.

Globally, trials were able to replicate high levels of aggregate intervention effectiveness for IIT, SIT or a combination of both strategies [3–14]. However, intervention effectiveness has been found to be heterogeneous in the same study settings, across spatial locations, such as directly treated areas, those at the borders, or those adjacent to treatment areas, driven potentially by mosquito migration [4]. Intervention effectiveness can also vary across time due to seasonality [4] and the time required for IIT-SIT to induce suppressive effects on wild-type mosquito populations. Changes in climate across sites over time may alter environmental carrying capacity over treatment locations and also affect both wild-type and *Wolbachia*-infected mosquitoes life history traits [15–17]. Lastly, across trials, the quality of evidence may be low, due to ad-hoc selection of a small number of control sites with potential differences in baseline characteristics from those of the intervention arm [6,9,10].

Evaluating the entomological effectiveness of IIT-SIT interventions primarily relies on measurements taken from mosquito traps (BG traps, ovitraps, Gravitraps), comparing the counts of eggs collected and/or the wild-type mosquito abundance on an aggregate basis in intervention versus control sites [4]. This can mask trap-level or location level heterogeneities in intervention effects and differences in intervention effects across time. Potentially, intervention effects could be driven by historical differences in trap-level mosquito abundance as well as anthropogenic and environmental factors [4]. In the post-intervention period, differences in suppression can be due to proximity to untreated areas; for example, (1) edge effects have been shown in a field trial of Singapore, where individual trap abundance was higher if it was closer to non-release locations versus if it was in the interior of the release site [4] (2) In a trial of the epidemiological effectiveness of mosquito-disseminated pyriproxyfen in Brazil, untreated zones close to intervention sites were conferred reductions in dengue incidence even though sites were not directly treated by intervention [18]. Understanding heterogeneities in treatment effects and the factors driving these heterogeneities can help advice optimization strategies for IIT-SIT release, and potentially confer performance gains in suppressive efficacy against target-species.

Therefore, the primary objectives of our study were 3-fold: We developed a causal-inference framework which explicitly accounts for spatio-temporal trap-level mosquito abundance data from 66,712 unique mosquito traps routinely maintained and measured to quantify (**1**) heterogeneous treatment effects for IIT-SIT across the time-since-intervention, over the traps’ historical mosquito abundance, over calendar time (**2**) whether elimination of wild-type *Ae. aegypti* was possible in intervention locations (**3**) if suppressive effects in locations adjacent to directly treated sites were heterogeneous, by exploiting the staggered adoption setting of the study setting.

## Methods

### Data

Entomological surveillance data was collected from Gravitraps which were designed to attract and catch gravid female *Aedes* mosquitoes. Each trap was measured twice a week, and can be individually geo-located to the postal code, representing a single apartment block. Surveillance data from Epidemiological Week (EW) 1 2019 to EW26 2022 was considered to evaluate the heterogeneous impact of *Wolbachia* IIT-SIT on wild-type *Aedes aegypti* and *albopictus* populations. Data after EW26 2022, was excluded due to the rollout of a large-scale randomised controlled trial in the study setting in most high-risk locations in Singapore, which made construction of the synthetic control donor pool challenging [19,20]. In this field study, four townships were selected to test two different intervention release strategies — an expanding release strategy and a targeted release strategy. The expanding release strategy is implemented by progressively expanding the size of release areas over time whereas the targeted release strategy is implemented in sites in interest. Yishun and Tampines were two large towns selected for the expanding release strategy, whereas Bukit Batok and Choa Chu Kang towns were selected for a targeted release approach^4^. The spatial resolution used for analyses was traps within sectors which comprise 10 – 20 public housing apartment blocks each and measure an average of approximately 0.088 km^2^. Sectors are used for the administration of surveillance and control for environmental infectious diseases in Singapore. To ensure that sufficient data was available to construct distributional synthetic controls, only sectors that had a pre-treatment period of 52 weeks or more were included, with 91 sectors included in the study as treated or intervened sectors. Similarly, the donor pool consisted of sectors that had never received the intervention before and during the study period, and were not adjacent to release sectors. To minimize the possibility of spillover contamination in control sectors comprising the donor pool, we only included control sectors which were non-adjacent to the intervention sectors for analysis. We included 662 sectors as potential control sectors in the donor pool. The average minimum distance of these control sectors to the nearest intervened sector was 4580.74 meters, which is far larger than the average flight distance (105.69 meters) of *Ae. aegypti* [21].

Releases of *Wolbachia*-infected male *Ae. aegyp*ti mosquitoes were conducted at fixed designated locations in the high-rise apartment blocks. There are 6–12 release locations which are equally distributed among ground level, middle floors (levels 5 – 6) and high floors (levels 10 – 11) of each apartment block. To maintain the density of intervention and reduce effect variation, *Wolbachia*-infected male mosquitoes were released twice a week in intervened sites. The density of release is uniform across sectors. Treatment for every sector started at different weeks and ended at the same week (EW26, 2022) in this study.

### Distributional synthetic control (DSC) method

We were motivated by prior studies [4,22] which revealed spatio-temporal variations in IIT-SIT intervention effectiveness, which may be driven by edge effects, spatial proximity of site to untreated/treated locations and intervention time. Prior work also used average mosquito abundance over time as the outcome variable, and the canonical synthetic control method to obtain intervention effectiveness. This ignored finer-scale trap-level information, which can inform how treatment effects are different across different traps in intervention sites, and whether mosquito elimination is possible under IIT-SIT. For example, the percentage of mosquito traps that have no mosquitoes recorded in intervention versus comparator sites can be assessed to ascertain whether the intervention led to mosquito elimination.

**Fig 1:**
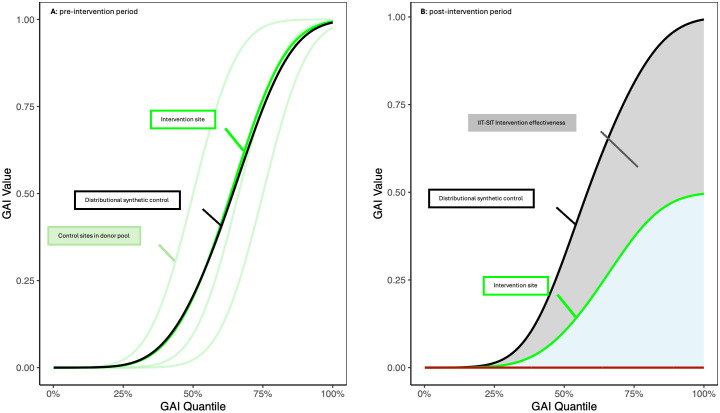
Illustration of distributional synthetic controls (A) optimizing weights to minimize the difference between a weighted average of quantile functions in the donor pool and the quantile function of the intervention sector in the pre-intervention period (B) taking differences between the quantile function of the DSC and intervention sectors to obtain the measure of intervention effectiveness. GAI, Gravitrap *Ae. aegypti* index. Lines represent the quantile functions of the control sites (in pale light green), the intervention site (in solid light green) and the DSC (in black).

We aimed to use the DSC method to understand heterogeneity in IIT-SIT treatment effects. Briefly, the DSC method aims to construct a counterfactual quantile function that characterises the quantile of mosquito abundances or the percentage of traps with no recorded mosquitoes in the intervention sector in the post-intervention period as though it did not receive treatment. The DSC for an intervention sector was obtained by optimizing weights which minimized the difference between a weighted average of quantile functions in the donor pool and the quantile function of the intervention sector in the pre-intervention period (Fig 1A). In the post-intervention period, the differences between the quantile function of the DSC and intervention sectors were taken to obtain the measure of intervention effectiveness across the observed quantiles of mosquito abundance between comparator groups (Fig 1B).

To obtain a sufficient number of observations to construct smooth quantile functions, we binned trap-level observations of mosquito abundance to each quarter (13 weeks) for each eligible sector. After binning, binned time intervals shorter than 13 weeks in pre-treatment period were removed from analysis. These observations were then used to construct continuous, quarterly, sector-level empirical quantile distributions $F^{-\text{1}}$ of the average trap-level number of trapped wild female *Ae. aegypti* mosquitoes for a specific postal code in that sector, which were subsequently smoothened using linear interpolation [23]. The default algorithm of estimating empirical quantile functions in the DSC framework was implemented for continuous variables. To obtain the optimal weights which minimized the differences between a weighted average of quantile functions in the donor pool and the quantile function of the intervention sector in the pre-intervention period, the 2-Wasserstein distance of quantile functions was computed as the objective function for optimization of continuous variables (e.g., mosquito abundance) and the 1-Wasserstein distance of cumulative density functions (CDF) was computed for optimization in discrete variables (e.g., mosquito elimination or not). The 2-Wasserstein distance $\hat{W_{\text{2}}}$ described differences between two quantile distributions $\overset{-\text{1}}{\underset{\text{1}}{F}},\;\overset{-\text{1}}{\underset{\text{2}}{F}}$ and was calculated discretely by equally dividing n=1000 points from x ϵ [0,1] and then computing based on trapezoidal rule:


$$\begin{array}{c} \hat{W_{\text{2}}}\left(\overset{-\text{1}}{\underset{\text{1}}{F}},\overset{-\text{1}}{\underset{\text{2}}{F}}\right)={\left(\frac{\text{1}}{n}\left(\sum_{x=\text{0}.\text{0}\text{0}\text{1}}^{\text{0}.\text{9}\text{9}\text{9}}{\left|\overset{-\text{1}}{\underset{\text{1}}{F}}(x)-\overset{-\text{1}}{\underset{\text{2}}{F}}(x)\right|}^{\text{2}}+\frac{{\left|\overset{-\text{1}}{\underset{\text{1}}{F}}(\text{0})-\overset{-\text{1}}{\underset{\text{2}}{F}}(\text{0})\right|}^{\text{2}}+{\left|\overset{-\text{1}}{\underset{\text{1}}{F}}(\text{1})-\overset{-\text{1}}{\underset{\text{2}}{F}}(\text{1})\right|}^{\text{2}}}{\text{2}}\right)\right)}^{\frac{\text{1}}{\text{2}}} \end{array}$$

The 1-Wasserstein distance $\hat{W_{\text{1}}}$ described the difference between two CDFs $F_{\text{1}},\;F_{\text{2}}$ and is calculated by summing cumulative discrete differences among ordinal variables y∈{1,2,…,Y}:


$$\begin{array}{c} \hat{W_{\text{1}}}\left(F_{\text{1}},F_{\text{2}}\right)=\sum_{y=\text{1}}^{Y}\left|F_{\text{1}}(y)-\;F_{\text{2}}(y)\right| \end{array}$$

The Jensen-Shannon divergence (JSD) was also used as another possible divergence metric for the discrete CDF, and is computed as:


$$\begin{array}{c} JSD\left(f_{\text{1}}\;||\;f_{\text{2}}\right)=\frac{\text{1}}{\text{2}}*\left(f_{\text{1}}\text{log}\left(\frac{f_{\text{2}}}{f_{m}}\right)\right)+\frac{\text{1}}{\text{2}}*\left(f_{\text{2}}\text{log}\left(\frac{f_{\text{1}}}{f_{m}}\right)\right) \end{array}$$

where $f_{m}$ is a mixture distribution of probability distribution $f_{\text{1}},\;f_{\text{2}}$ calculated by $f_{m}=\frac{f_{\text{1}}+f_{\text{2}}}{\text{2}}$. Since there were 4–12 pre-treatment time intervals in the treated sectors, large variance on weights across pre-treatment periods would be expected if optimized weights were calculated separately from each pre-treatment interval. To alleviate this, we optimized the weights of control sectors only once by minimizing the sum of objective functions in all binned time intervals in the pre-treatment period.

As the donor pool contained a large number of control sectors, most sectors comprising the donor pool would assign a null weight value on the construction of DSCs. To reduce the computational load, we first empirically computed the similarity in distributions by computing the correlation in mosquito abundance between control and intervention sectors in the pre-intervention period. Correlation is defined based on the average 2-Wasserstein distance for analysing mosquito abundance and the average Jensen-Shannon divergence for analysing the percentage of traps recording zero mosquitoes (i.e., elimination) between two sectors over all time intervals in the pre-intervention period. Only the top 40 control sectors which were most correlated to a specific intervention sector were used for fitting the DSC for a specific intervention sector (Fig J in S1 File).

### Accounting for discrete values in trap-level measurements

Gravitrap abundance index (GAI) represents the mosquito abundance at designated locations, calculated as the average number of wild female *Ae. aegypti* over the number of functional Gravitraps across the pre-specified spatio-temporal scale. At the trap-level, recorded abundances are at discrete 0.5 value intervals. We explored (**1**) building discrete quantile functions by trap for each sector, and (**2**) aggregating GAI values by postal code to convert GAI values from discrete to continuous ones. Plots were compared by visual inspection and the smoother representation of mosquito abundance was chosen.

We used the procedure of Li and Tolhurst by replacing uniform random sampling of quantile functions with beta distribution random sampling [24]. This was done to reduce the negative sampling effect of extreme values. As the data consisted more pre-intervention time periods and potentially extreme values in the upper tail of some quantile functions, the beta (3,3) distribution was used as the sampling method. Minimizing the sum of 2-Wasserstein distances between quantile functions of the treated sector *d* and control sectors *j* among pre-intervention time intervals was considered to obtain optimal DSC weights $\lambda_{d}=(\lambda_{d\text{1}},\;\lambda_{d\text{2}},\ldots ,\lambda_{dj},\ldots ,\lambda_{dJ})$ for the intervened sector *d* across the whole pre-intervention period $t<T_{\text{0}}$ and the objective function for determining the DSC weights was as follows:


$$\begin{array}{c} \lambda_{d}=\underset{\lambda_{dj}}{\text{argmin}}\left[\sum_{t=\text{1}}^{T_{\text{0}}-\text{1}}{\hat{W}}_{\text{2}}\left(\overset{-\text{1}}{\underset{d,t}{F}},\sum_{j=\text{1}}^{J}\lambda_{dj}\overset{-\text{1}}{\underset{j,t}{F}}\right)\right] \end{array}$$

where $\overset{-\text{1}}{\underset{d,t}{F}}$ denotes the quantile function for mosquito abundance at intervened sector *d* at time interval *t*, $\overset{-\text{1}}{\underset{j,t}{F}}$ the quantile function for mosqui*t*o abundance at control sector *j* at time interval *t*, $T_{\text{0}}$ the treatment start week, $\lambda_{dj}$ the weigh*t* for control sector *j* for the corresponding intervened sector *d*. The counterfactual quantile distribution of mosquito abundance $\overset{-\text{1}}{\underset{cf,d,t}{F}}$ at an intervened sector *d* in post-intervention time intervals $t\geq T_{\text{0}}$ was computed as follows:


$$\begin{array}{c} \overset{-\text{1}}{\underset{cf,d,t}{F}}=\lambda_{d}\cdot (\overset{-\text{1}}{\underset{\text{1}t}{F}},\ldots ,\overset{-\text{1}}{\underset{Jt}{F}})=\;\sum_{j=\text{1}}^{J}\lambda_{dj}\overset{-\text{1}}{\underset{j,t}{F}} \end{array}$$

### Examining the possibility of wild-type mosquito elimination

To examine whether wild-type elimination of mosquitoes was possible under IIT-SIT, we rehashed our outcome to be the percentage of traps recording zero mosquitoes (instead of mosquito abundance). The cumulative density function was therefore taken as:


$$\begin{array}{c} F(x)=\left\{ \begin{array}{c} p(\text{0}),\;x\;\epsilon \;[\text{0},\text{1})\;\;\;\;\;\;\;\;\;\;\;\;\\ \text{1},\;x=\text{1} \end{array}\right.\text{ from }f(x)=\left\{ \begin{array}{c} \;\;\;\;\;\;\;\;\;\;\;\;p(\text{0}),\;x=\text{0}\;\\ \text{1}-p(\text{0}),\;x=\text{1} \end{array}\right.\; \end{array}$$

where *x* denotes two possible statuses in a trap: x=0 denotes no *Aedes aegypti/albopictus* recorded and x=1 otherwise. This representation always leaves F(x)=1 for x=1, which simplifies the estimation of counterfactual CDF from fitting two F(0) and F(1) to one parameter F(0).

Given the CDF, we tested the use of the (**1**) 1-D Wasserstein distance and (**2**) Jensen-Shannon divergence to be applied as objective functions to find the optimal weight to construct the sector’s distributions during pre-treatment period. The 1-D Wasserstein distance between the actual distribution in the intervened sector *d* and the distributional synthetic control is:


$$\begin{array}{c} \hat{W_{\text{1}}}\left(\lambda_{jt}F_{j,t},F_{d,t}\right)=\sum_{y}\left|\sum_{j=\text{1}}^{J}\lambda_{jt}F_{j,t}(y)-\;F_{d,t}(y)\right|=\left|\sum_{j=\text{1}}^{J}{\lambda_{jt}f}_{j,t}(\text{0})-\;f_{d,t}(\text{0})\right| \end{array}$$

where the second equality holds as there are only two possible values for the outcome *y*. Whereas JSD is computed as


$$\begin{array}{c} JSD_{convex}\left(F_{d,t}\;||\;F_{cf,d,t}\right)=\frac{\text{1}}{\text{2}}*\left(F_{d,t}\text{log}\left(\frac{F_{d,t}}{F_{mt}}\right)-F_{d,t}+F_{mt}\right)+\frac{\text{1}}{\text{2}}*(\;F_{cf,d,t}\text{log}\left(\frac{F_{cf,d,t}}{F_{mt}}\right)-F_{cf,d,t}+F_{mt}) \end{array}$$

where ${\;F}_{d,t}$ denotes the CDF for the intervened sector *d* at time *t*, $F_{cf,d,t}=\;\sum_{j=\text{1}}^{J}\lambda_{jt}F_{j,t}$ denotes the counterfac*t*ual CDF by optimal weights and control sector CDFs at time *t*, $F_{mt}=(F_{d,t}+\;F_{cf,d,t})/\text{2}$ denotes a mixture function of $F_{d,t}$ and $F_{cf,d,t}$ at time *t*. $JSD_{convex}$ here deviates from *t*he prior JSD definition due to convexity and is only applied in the weight optimization step.

We used the entire pre-intervention period to compute objective functions and find optimal weights which comprised the distributional synthetic controls. As preliminary analyses comparing the differences of each fitted distribution and the original distribution by both 1-Wasserstein distance score and the JSD showed that most of the treated sectors were fitted better using the 1-Wasserstein distance method versus the JSD, the 1-Wasserstein distance method was applied as the objective function in weight selection (Fig T in S1 File). The optimal weights for each sector’s DSC were $\lambda_{elimination,d}=\left(\lambda_{d\text{1}},\;\lambda_{d\text{2}},\ldots ,\lambda_{dJ}\right)$ and was estimated by:


$$\begin{array}{c} \lambda_{elimination,d}=\;\underset{\lambda_{dj}}{\text{argmin}}\sum_{t}\left|F_{d,t}-\;\sum_{j=\text{1}}^{J}\lambda_{dj}F_{j,t}\right|=\underset{\lambda_{dj}}{\text{argmin}}\sum_{t}\left|f_{d,t}(\text{0})-\;\sum_{j=\text{1}}^{J}\lambda_{dj}f_{j,t}(\text{0})\right| \end{array}$$

where $t<T_{\text{0}}$. Counterfactual CDFs of mosquito elimination $F_{cf,d,t}$ at an intervened sector *d* in post-intervention time intervals $t\geq T_{\text{0}}$ were computed as follows:


$$\begin{array}{c} F_{cf,d,t}=\lambda_{elimination,d}\cdot (F_{\text{1},t},\ldots ,F_{J,t})=\sum_{j=\text{1}}^{J}\lambda_{dj}F_{j,t} \end{array}$$

### Estimating heterogeneous intervention effectiveness

Intervention effectiveness (IE) was computed for both directly treated intervention and adjacent spillover sectors. For directly treated sectors, we computed IEs by core or buffer sectors. Buffer sectors were directly treated sectors which shared borders with untreated sectors and core sectors were directly treated sectors which did not share borders with untreated sectors. Adjacent-spillover sectors were defined as never-treated or not-yet-treated sectors that shared geographical boundaries with treated sectors. Once an adjacent spillover sector adopted treatment, the data after treatment was not used to evaluate spillover IE. DSCs were computed for adjacent spillover sectors the same way as directly treated locations, and the donor pool consisted of only non-treated sectors never adjacent to a treated sector. We aggregated IEs by these sector categories by adoption period and geographical configurations over the post-intervention period. We aggregated IEs by calendar time, and by event-time, that is, the time since intervention was adopted in each location. Respectively, these aggregations helped quantify if there were temporal differences in the reductions of mosquito abundance or increased likelihood of wild-type elimination over the time of the study, or over the time since interventions were adopted. The same analytical procedures were used to examine the impact of IIT-SIT on *Ae. albopictus abundance*.

We also analysed the possibility of mosquito elimination under IIT-SIT, where IE was computed by substituting CDFs. Lastly, raw reductions (rather than relative reductions) in mosquito abundance were computed by taking the difference in quantiles between the observed GAI distribution and the counterfactual distribution estimated by the DSC.

Current DSC literature only proposed quantifying treatment effects via raw quantile value differences. This represented raw rather than relative treatment effects. We introduced a baseline scenario of no mosquitoes being detected, so that it can be scored against the mosquito abundance for a sector. This allowed us to calculate 2-Wasserstein distances between the GAI distributions at the sector and the baseline scenario. To compute IEs, we took the differences in scores of the intervened location’s mosquito abundance and the distributional synthetic control to the scenario of wild-type mosquito elimination ($\overset{-\text{1}}{\underset{perfect}{F}}$). In examining the effect of IIT-SIT on mosquito abundance, the quantity expresses the relative differences in distances between the treated/counterfactual location to the scenario of null wild-type mosquito abundance ${(f}_{perfect}(\text{0})=\text{1})$. For a specific sector *d*, the IE at time *t* was calculated as follow:


$$\begin{array}{c} IE_{d,t}=\;\frac{\hat{W_{\text{2}}}\left(\overset{-\text{1}}{\underset{cf,d,t}{F}},\;\overset{-\text{1}}{\underset{perfect}{F}}\right)-\;\hat{W_{\text{2}}}(\overset{-\text{1}}{\underset{d,t}{F}},\;\overset{-\text{1}}{\underset{perfect}{F}})\;}{\hat{W_{\text{2}}}(\overset{-\text{1}}{\underset{cf,d,t}{F}},\;\overset{-\text{1}}{\underset{perfect}{F}})} \end{array}$$

where ${F_{perfect}}^{-\text{1}}$ is a degenerate distribution concentrated at zero with ${F_{perfect}}^{-\text{1}}(q)=\text{0},\;q\;\epsilon \;[\text{0},\text{1}]$ and characterizes the scenario with zero mosquitoes in the sector. The IE has a range (−∞,1]. When the actual mosquito abundance in treated sectors is near the scenario of mosquito elimination, IE ≈ 100%. When the observed mosquito distribution is further from the scenario of mosquito elimination versus the counterfactual distribution as estimated using the distributional synthetic control framework, IE is lower than 0. A protective treatment effect should have IE in the range of (0,1]. As the distributions of mosquito abundance were summarized by binning across consecutive 13-week periods (e.g., weeks 1 – 13, 14 – 26 in post-intervention period), $IE_{d,t}$ kept constant within each 13-week interval, and the average 13-week IE was calculated as the mean of $IE_{d,t}$ among sectors *d* which had surveillance data across the 13-week interval:


$$\begin{array}{c} IE_{t}=\frac{\text{1}}{n_{t}}\sum_{d}IE_{d,t} \end{array}$$

where $n_{t}$ is the number of sectors which had surveillance data across the 13-week interval. Whereas in examining the possibility of wild-type mosquito elimination in IIT-SIT treated sectors, we computed the increase in the percentage of traps which do not record mosquitoes as the difference in the percentage of traps which do not record mosquitoes in the intervention sector and its corresponding counterfactual sector. We aggregated this quantity by calendar time and time-since-intervention.

We also directly computed raw reductions in mosquito abundance in the post-intervention period by differencing the counterfactual and observed quantile functions of mosquito abundance for intervened sectors. For a specific post-intervention time interval $t\geq T_{\text{0}}$, empirical quantile-based raw reduction function $G_{t}$ was calculated as follows:


$$\begin{array}{c} G_{t}(q)=\frac{\text{1}}{n_{t}}\sum_{d}\overset{-\text{1}}{\underset{cf,d,t}{F}}(q)-\overset{-\text{1}}{\underset{d,t}{F}}(q) \end{array}$$

where q∈{0,0.001,…,0.999, 1} are discrete quantiles, $n_{t}$ is number of intervened sectors with mosquito abundance recorded at time *t*, $\overset{-\text{1}}{\underset{d,t}{F}}$ is the actual quantile function for intervened sector *d* at time *t*, and $\overset{-\text{1}}{\underset{cf,d,t}{F}}$ is counterfactual quantile function for intervened sector *d* at time *t*. Quantile-based IE was also computed to quantify treatment effect heterogeneity, estimating the IEs in each post-interven*t*ion time interval across a specific area. This was computed as:


$$\begin{array}{c} IE_{quantile,t}(q)=\frac{\text{1}}{n_{t}}\sum_{d}\frac{\left(\overset{-\text{1}}{\underset{cf,d,t}{F}}(q)-\overset{-\text{1}}{\underset{perfect}{F}}(q))-(\overset{-\text{1}}{\underset{d,t}{F}}(q)-\overset{-\text{1}}{\underset{perfect}{F}}(q)\right)}{\overset{-\text{1}}{\underset{cf,d,t}{F}}(q)}=\;\frac{\text{1}}{n_{t}}\sum_{i}\frac{\overset{-\text{1}}{\underset{cf,d,t}{F}}(q)-\overset{-\text{1}}{\underset{d,t}{F}}(q)}{\overset{-\text{1}}{\underset{cf,d,t}{F}}(q)} \end{array}$$

95% confidence intervals (CI) of average IE and raw reduction were calculated via bootstrap following Dijcke, Gunsilius and Wright [25]. The bootstrap procedure was employed because the statistics are non-linear or have unknown distributions. For each pre-treatment interval, trap-level measurements in control sectors were resampled with replacement, and new empirical quantile functions $\overset{-\text{1}}{\underset{j,t}{\tilde{F}}}$ were estimated. New counterfactual quantile functions $\overset{-\text{1}}{\underset{cf,d,t}{\tilde{F}}}$ were constructed based on a new weighting scheme for the intervened sector. The resampling was repeated 500 times and the statistics in interest were repeatedly estimated. We obtained confidence intervals via the percentile method, where the 2.5^*th*^ and 97.5^*th*^ quantile were used as the lower and upper bound of the 95% CI.

### Robustness checks

We conducted a large battery of robustness checks to confirm the internal validity of our analytical procedure. (**1**) We conducted in-time placebo checks, where we re-ran our analytical procedure and considered pseudo-intervention start points 26 and 52 weeks before actual treatment start dates, to see if there were any pre-trends in mosquito abundance which confounded our study (**2**) We conducted in-space placebo checks, where we re-ran our analytical procedure on control sectors which never received treatment, and assigned pseudo-interventions to these control sectors. This was done to see if spurious intervention effects were detected in control sectors. (**3**) We conducted visual inspection checks on all fitted DSCs to see if model fit to actual quantile functions in the intervention sectors were acceptable in the pre-intervention period (**4**) In addition to the bootstrap inference procedure, we conducted the placebo permutation test, where every control sector was reassigned to become pseudo-intervened sector to estimate its post-intervention effect, from which p-values could be obtained from the percentage of sectors that had an IE not smaller than the treated sector to assess statistical significance.

## Results

We included a total of 7,819, 3,255 and 51,343 trap measurements in directly-treated, spillover and control sectors for analysis after exclusion. The total number of trap-measurements and sectors evaluated across the study period was provided in Table 1.

**Table 1:** Number of traps and study sectors (in brackets) used for analysis.

	EW8 – EW26 2020	EW27 – EW53 2020	EW1 – EW26 2021	EW27 – EW52 2021	EW1 – EW26 2022	Total
Sites	Traps (Sectors)					
Core^1^	549 (7)	1144 (13)	2016 (23)	2555 (33)	5358 (69)	5520 (69)
Buffer^2^	2205 (26)	3148 (37)	2843 (37)	2798 (36)	2795 (34)	4488 (50)
Direct^3^	2567 (31)	3902 (46)	4402 (55)	5066 (64)	7287 (91)	7819 (91)
Spillover^4^	1949 (23)	2118 (28)	1988 (25)	1672 (21)	1531 (20)	3255 (40)
Control^5^	45670 (662)	45799 (662)	46688 (662)	46280 (662)	46253 (662)	51343 (662)

^1^Directly-treated sectors which did not share borders with control sectors.

^2^Directly-treated sectors which shared borders with control sectors.

^3^Directly treated sectors.

^4^Spillover sectors refer to locations which shared borders with treated sectors but were not directly treated.

^5^Control sectors which do not share borders with directly treated sectors and are not treated.

*Note that traps in a sectors may be counted in both “Core” and “Buffer” sites when the treated sectors changed from being designated a buffer to core sector during that specific time interval.

### Intervention effectiveness in release sectors increased with intervention time

**Fig 2:**
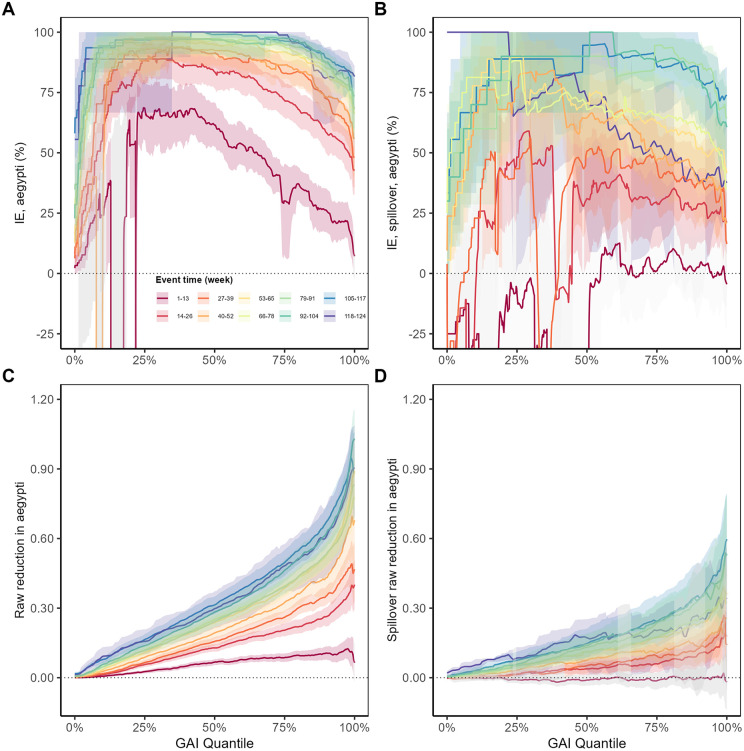
(A) IIT-SIT intervention effectiveness to reduce *Ae. aegypti* abundance by time-since-intervention in directly intervened sectors by GAI quantile. **(B)** IIT-SIT intervention effectiveness to reduce *Ae. aegypti* abundance by time-since-intervention in spillover sectors by GAI quantile. **(C)** Raw reduction distribution in *Ae. aegypti* abundance by time-since-intervention in directly intervened sectors. **(D)** Raw reduction distribution in *Ae. aegypti* abundance by time-since-intervention in spillover intervened sectors. The coloured lines represent the average values across the sectors. The shaded regions represent the pointwise 95% confidence intervals. The shaded regions in grey represent quantiles where there is an insignificant reduction in *Ae. aegypti* populations due to intervention. The shaded regions in other colours represent quantiles where there is a significant reduction in *Ae. aegypti* populations due to intervention. Plots **(A)**, **(B)** have been truncated as the intervention effectiveness estimates are negative and have 95% CIs which are not bounded by 0.

By comparing the quantile functions between intervention sectors and the respective distributional synthetic controls, we found that the direct intervention effectiveness (IE) was greater in locations that were subject to longer intervention periods (Fig 2A). From 1 – 52 weeks post intervention, the average 13-week IE ranged from 24.04% (95% CI: 16.61% – 30.85%) to 71.51% (95% CI: 65.02% – 77.18%). In contrast, 53 – 124 weeks post intervention, the average 13-week IE ranged from 80.04% (95% CI: 76.26% – 83.46%) to 86.08% (95% CI: 82.89% – 88.78%). Raw reductions in abundance were also greater in locations that were subject to longer intervention periods (Fig 2C).

By comparing the IEs over different quantiles, we found that in directly treated sectors, quantile IEs around the 25^*th*^ quantile were higher than those at other quantiles. Quantile IEs around the 0^*th*^ quantile were lower and gradually increased as quantiles increased. Comparing quantile IE between the 25^*th*^ – 49.9^*th*^ quantile and 75^*th*^ – 100^*th*^ quantile, the maximum quantile IE difference decreased from 61.33% in 1 – 13 weeks after intervention to 29.62% in week 105 – 117, and the minimum quantile IE difference decreased from 24.40% to −0.7%. This showed that IE reduced quickly at higher GAI quantiles at the beginning of treatment, and the treatment effect was lower in moderate mosquito abundance areas in the sector. Longer treatment times could, however, shrink the difference in treatment effectiveness between mosquito quantiles. Though the IE reduced with higher quantile of GAI, the raw reduction increases with higher quantiles (Fig 2A and 2C).

IE in later study periods (EW1 – 26, 2022) was also higher than that in earlier study periods (EW8 – 53, 2020). Mean relative reductions in *Ae. aegypti* abundance were heterogeneous in the initial study period (i.e., earlier e-weeks) (Fig A in S1 File), but gradually converged at around 1-year post intervention with more than 80% suppressive effectiveness on *Ae. aegypti* abundance (Fig 1A).

We also computed the average IE between core sectors and buffer sectors. We did not find significant IE differences across the two-year post-intervention period (Fig B in S1 File).

### Intervention effectiveness in spillover sectors increased with intervention time

A similar pattern was found in spillover locations which shared geographical borders with direct intervention sectors. Spillover IE in *Ae. aegypti* abundance increased with increasing intervention duration (Fig 2C). From 1 – 52 weeks post intervention, the average spillover IE ranged from −1.29% (95% CI: −14.67% – 10.24%) to 37.98% (95% CI: 25.07% – 49.42%). In contrast, 53–117 weeks post intervention, the average spillover IE ranged from 52.65% (95% CI: 42.72% – 61.99%) to 78.08% (95% CI: 72.36% – 83.24%). We observed a decrease in IE in the 118 – 124 weeks, which may be due to the insufficient number of sectors in that period required for valid estimates of intervention effectiveness (n = 3). Raw reductions in abundance were also greater in spillover locations that were subject to longer intervention periods (Fig 2D). The result indicated a negative but insignificant intervention effectiveness estimate in sites defined to be spillover locations at the start of the trial. This potentially reflects sampling noise and migration of wild-type mosquitoes at earlier treatment times.

Comparing IE between directly intervened sectors and spillover sectors across intervention time, average IE in intervened sectors was higher versus adjacent, non-treated sectors in most quantiles (Fig 2A, 2B). We also found that raw GAI reductions in spillover sectors were consistently lower than those directly intervened versus across all quantiles over the intervention period (Fig 2C, 2D).

### Spatial and temporal impact of intervention

**Fig 3:**
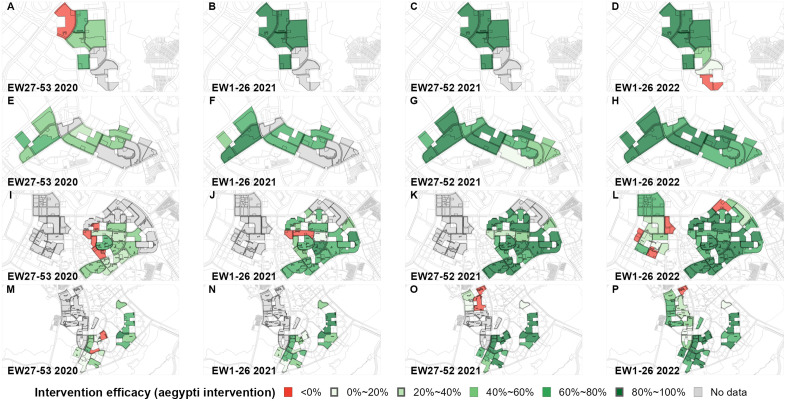
Spatio-temporal configuration of intervention effectiveness of IIT-SIT on *Ae. aegypti* abundance in treated sectors. Columns represent the respective time periods of EW27 – 53 2020, EW1 – 26 2021, EW27 – 52 2021 and EW1 – 26 2022. Rows represent each township. From top to bottom: Bukit Batok, Choa Chu Kang, Tampines and Yishun townships are represented. Base layer from the Master Plan 2019 Land Use Layer dataset, licensed under the Singapore Open Data Licence version 1.0. https://data.gov.sg/datasets?query=master+plan+2019+land+use+layer.

Reductions in *Ae. aegypti* abundance were consistent and converged to a high-level, with the mean $IE_{d,t}$ being around 65.06% near the end of the 26-week study period. This was consistent across sectors and townships, and aligned with previous study results (Fig 3).

### Intervention impact by historical abundance and by township

**Fig 4:**
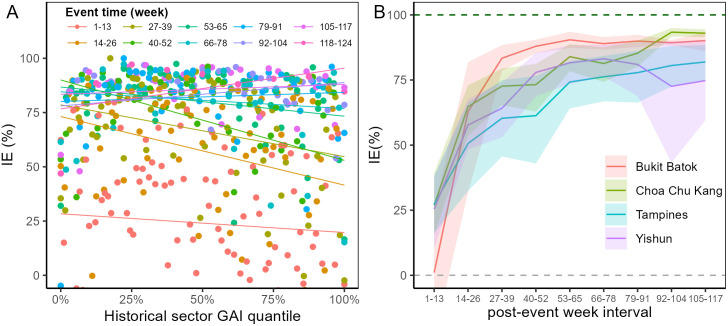
(A) Sector intervention effectiveness of IIT-SIT to reduce *Ae. aegypti* abundance over previous sector relative *Ae. aegypti* abundance quantile in direct intervened sectors. Coloured lines represent GAM models based on corresponding post-intervention IE. Each dot represents a unique sector evaluated at that specific event-time. **(B)** Mean intervention effectiveness of IIT-SIT to reduce in *Ae. aegypti* abundance in direct intervened sectors by township. Coloured lines represent average IE across the sectors in each township. The shaded areas represent pointwise 95% confidence intervals.

$IE_{d,t}$ was significantly higher in sectors with lower historical pre-treatment GAI. However, as the intervention time increased, IEs over different historical mosquito quantiles converged to a similar level (Fig 4A). Intervention effectiveness increased with more intervention time, with little heterogeneity across intervened townships (Fig 4B). Areas with historical high mosquito populations required 52 weeks to reach 75% suppression, while areas with low historical mosquito populations required 26 weeks.

### Examining the potential for wild-type *Ae. aegypti* elimination

**Fig 5:**
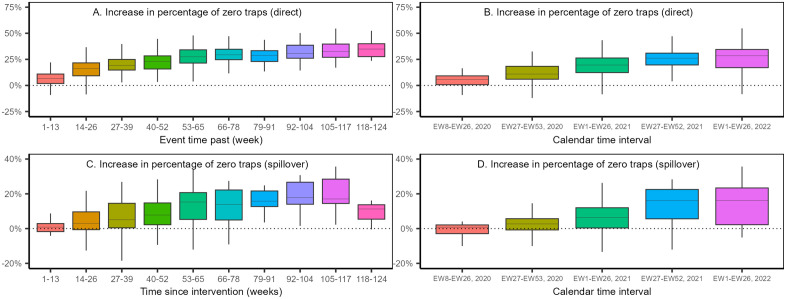
(A) Increase in the percentage of traps recording no mosquitoes by time-since-intervention in directly intervened sectors. **(B)** Increase in the percentage of traps recording no mosquitoes by study time in directly intervened sectors. **(C)** Increase in the percentage of traps recording no mosquitoes by time-since-intervention in sectors located adjacent to directly intervened sectors. **(D)** Increase in the percentage of traps recording no mosquitoes by study time in sectors located adjacent to directly intervened sectors. Lines in the boxes represent the middle 50% of the estimate, whereas the ends of the boxes represent the interquartile range, spanning from the 25^th^ percentile to the 75^th^ percentile. The lines represent the minimum and maximum respectively.

We further analysed if the intervention led to an increase in the percentages of traps recording no wild-type female *Ae. aegypti* mosquitoes. This was done to ascertain if the elimination of wild-type female *Ae. aegypti* mosquitoes was possible given sufficient intervention time. We found that the mean increase in the percentages of traps recording no wild-type female *Ae. aegypti* mosquitoes was around 6.77% (95% CI: 5.49% – 8.12%) – 33.01% (95% CI: 29.50% – 36.57%) in the 1 – 124 weeks after interventions began (Fig 5A). In later trial periods, this increase was consistently high at 23.05% (95% CI: 20.23% – 25.78%) – 23.35% (95% CI: 20.68% – 26.16%) from 2021 EW27–2022 EW26 (Fig 5B). In spillover locations, the effect was found to be weaker (Fig 5C–5D), from 3.75% (95% CI: 0.84% – 7.08%) at 14 – 26 weeks post intervention to 18.81% (95% CI: 11.28% – 26.46%) at 105 – 117 weeks post-intervention.

### Robustness checks

All robustness checks are reported in detail in the supplementary information section (S1 File).

## Discussion

By using distributional synthetic controls, while we found that IIT-SIT interventions can lead to dramatic suppressive effects of up to 86.08% on adult *Aedes aegypti* abundance, we found that these interventions did not lead to mosquito elimination after 2–3 years of release. While the percentages of sectors with traps recording no mosquitoes increased (Fig 5A, 5C), after 52 weeks of intervention, this increase in the number of such traps only managed to stabilized at around 30% (Fig 5C). This may be due to the migration of wild-type vectors from non-release locations which interfere with the estimated suppressive effect of the intervention over a contiguous urban landscape. Cryptic populations of wild-type mosquitoes that cannot be reached by the intervention may also attenuate the possibility of elimination. However, these factors are likely to diminish given increasingly larger and longer rollouts of IIT-SIT across the city-state.

Furthermore, while IE was consistently high, it dipped in higher GAI quantiles, even though raw reductions increased consistently with higher GAI quantiles. We also found that IE was higher in sectors with lower historical GAI (Fig 4A), though IE converged across sectors with different historical GAI values as intervention time increased. Sectors with high historical abundance may reflect ecological conditions that are more favourable for wild-type mosquito populations, and may require a higher intensity of release or longer duration of releases to generate the same levels of suppressive effectiveness. As our study comprised evaluations of a short field trial period, intervention effectiveness may eventually converge for these historically high abundance locations once the suppressive effectiveness outpaces wild-type population growth. This also highlights the potential need to integrate *Wolbachia*-based suppression with other conventional control measures, such as environmental management and source reduction, to further reduce wild-type *Ae. aegypti* populations.

Raw reductions in *Ae. aegypti* abundance were also found to increase with more time spent in intervention sectors. In spillover sectors, IE was lower in magnitude (IE range: −1.29% – 78.08%), with raw reductions in *Ae. aegypti* abundance also found to increase with more time spent in spillover intervention sectors (Fig 2C). Estimates suggest that increasing immigration of *Wolbachia* mosquitoes into adjacent non-release sectors over the course of the intervention period can subsequently result in suppression of wild-type *Ae. aegypti* mosquitoes in adjacent non-release sectors. Significant intervention effectiveness in spillover sectors also highlights that not-yet-treated sectors may already be benefiting from spillover effects prior to direct release, which possibly contributed to higher direct IE during the later study periods (EW1 – 26, 2022) being higher than that of earlier study periods (EW8 – 53, 2020).

IE estimates yielded by DSC indicated treatment effect heterogeneity on both spatial and temporal scales. In contrast, the canonical SCM yielded pointwise IE estimates and showed temporal heterogeneity of treatment effects. In the canonical SCM, the intervention effectiveness of *Wolbachia* is evaluated via average mosquito abundance in sectors over time. However, this disregards the finer-scale trap-level information in sectors. Whereas DSC yields intervention effectiveness estimates using the entire quantile of observed mosquito abundance within sectors, which contains more information about the variation and spread of data in evaluated sectors. By obtaining counterfactual quantile functions as the comparator group to the observed quantile function in intervention sites, IE estimates can be expressed in other statistics, such as the IE over different mosquito abundance quantiles, and help tease out heterogeneous treatment effects over smaller spatial scales.

Our study has several strengths. Our analysis relied on a large, nationwide, routinely collected surveillance database on mosquito abundance. By using trap-level records, we could obtain and construct DSCs, which enabled us to understand the heterogeneous effects of *Wolbachia-Aedes* on wild-type *Aedes* abundance, and understand if the elimination of wild-type mosquitoes was possible. This analysis was supported by extensive internal validity checks – including in-space, in-time placebo checks, and additional inferential procedures such as permutation testing. These procedures enabled us to allay concerns that estimates of intervention effectiveness were spurious or due to poor model predictive performance. There are, however, certain limitations. Despite the extremely high resolution of our data, we tagged trap-level measurements into 13-week intervals at the sector level, which enabled us to understand heterogeneous intervention effectiveness across sites, but does not allow us to exactly identify the exact traps locations where wild-type mosquito populations do not have high IEs or elimination. Due to the short study period and initiation of a large-scale cluster randomised controlled trial in 2022 [19,20], further evaluation of entomological effectiveness in historically high-abundance locations, which have lower intervention effectiveness, cannot be done. In future studies, increasing the number of samples via more intensive surveillance can also enable analyses at a finer temporal resolution and potentially help DSC estimate finer-scale treatment effects. Lastly, the construction of DSCs assumed that non-intervened locations which were not adjacent to intervention sectors did not experience partial interference from intervention sectors. Yet, DSC relies on the stable unit treatment value assumption (SUTVA), requiring that the donor pool not be affected by treatment effects. This assumption is violated when the intervention generates spillover effects in the donor pool, which may lead to conservative intervention effectiveness estimates. While previous studies have shown limited dispersal for the *Ae. aegypti* vector in Singapore [26], interference of control sites may bias estimated intervention effectiveness downwards.

## Conclusion

This study provides strong evidence that IIT-SIT can drastically suppress wild-type *Aedes aegypti* populations despite heterogeneous treatment effects over time and space. Future work should seek to triangulate the factors driving incomplete mosquito elimination in intervention locations.

## Supporting information

S1 File Supplementary information comprising additional study results and internal validity checks for the analytical framework. A combination of texts, figures and tables describing (1) multiple robustness checks and sensitivity analyses and (2) additional study findings.
(DOCX)
