# Supplementary material for: Heterogeneous suppressive effect of Wolbachia incompatible insect technique coupled with sterile insect technique across time and historical Ae. aegypti abundance - using distributional synthetic controls
Source: PLoS Comput Biol. 2026 Jun 22;22(6):e1014355. doi: 10.1371/journal.pcbi.1014355 (PMC13336471; doi:10.1371/journal.pcbi.1014355)
Supplement: S1 File — A combination of texts, figures and tables describing (1) multiple robustness checks and sensitivity analyses and (2) additional study findings. (DOCX) [file pcbi.1014355.s001.docx]

**Impact of IIT-SIT on *Ae. aegypti* populations**

IE during the later study periods (EW1 – 26, 2022) was higher than that in earlier study periods (EW8 – 53, 2020) (Fig A2). Mean relative reductions in *Ae. aegypti* abundance were heterogenous in the initial study period (i.e. earlier e-weeks) and earlier timepoints that a trap location is intervened (Fig A2), but gradually converged at around 1-year post intervention with more than 80% suppressive effectiveness on *Ae. aegypti* abundance.

We further studied possible treatment effect heterogeneity by comparing IE over the intervention time between buffer and core sectors (Fig B1). However, we found similar trajectories in treatment effect. During the first 52-week treatment, average IE in core areas increased from 28.09% (95%CI: 17.78% – 40.81%) to 62.63% (95%CI: 48.41% – 81.78%), while average IE in buffer areas increased from 20.71% (95%CI: 11.52% – 29.65%) to 80.00% (95%CI: 75.76% – 84.54%). However, these effects were not different statistically (Welch’s T-test p-val >0.05).

IE in both buffer and core sectors increased over the study period (Fig B2). However, due to the staggered adoption of treatment, sectors in buffer zones received longer treatment times compared to those in core zones and therefore stronger treatment effects in the same calendar time.****

**Fig A**

1. Raw reduction distribution in *Ae. aegypti* abundance by calendar time in directly intervened sites
2. Quantile of IIT-SIT intervention effectiveness to reduce *Ae. aegypti* abundance by calendar time in directly intervened sites. The arrows indicate whiskers truncated at the plot boundary.

**Fig B**

1. IIT-SIT intervention effectiveness to reduce *Ae. aegypti* abundance by time-since-intervention in core versus buffer areas
2. IIT-SIT intervention effectiveness to reduce *Ae. aegypti* abundance by calendar time in core versus buffer areas

The coloured lines represent the average intervention effectiveness across the sectors in the buffer or core area. The shaded regions represent the pointwise 95% confidence intervals.

**Impact of IIT-SIT on *Ae. albopictus* populations**

We assessed if IIT-SIT had heterogenous impact on *Ae. albopictus* populations. There was no impact of IIT-SIT on *Ae. albopictus* populations at the 0^th^ – 24.9^th^ quantile (Fig C1,2). However, at larger quantiles, we estimated a small overall increase in *Ae. albopictus* populations, which expanded in magnitude as more time was spent in intervention sites. (Fig C1). In spillover locations, we found no significant change in *Ae. albopictus* populations over all quantiles (Fig C5). Across the study period, there was a slight increase in *Ae. albopictus* populations at the 75^th^ –100^th^ quantile (Fig C6). There were no significant differences in $IE_{d,t}$ between core and buffer areas during study period (Fig C10). Mean IE in direct-intervened townships showed a high absolute $IE_{d,t}$(−492.78%– −74.09%) 14 – 39 weeks post-intervention, and dropped to around −123.19% – −9.74% near the end of study period (Fig D2). Directly-treated sector $IE_{d,t}$ was higher in sites with lower historical *Ae. albopictus* abundance (Fig D1).

**Fig C**

(**1**) Cumulative distribution of raw reductions in *Ae. albopictus* abundance by time-since-intervention in directly intervened sites

(**2**) Cumulative distribution of reductions in *Ae. aegypti* abundance by calendar time in directly intervened sites

(**3**) Quantile of IIT-SIT intervention effectiveness to reduce *Ae. albopictus* abundance by time-since-intervention in directly intervened sites

(**4**) Quantile of IIT-SIT intervention effectiveness to reduce *Ae. albopictus* abundance by calendar time in directly intervened sites

(**5**) Cumulative distribution of raw reductions in *Ae. albopictus* abundance by time-since-intervention in sites adjacent to directly treated areas

(**6**) Cumulative distribution of reductions in *Ae. albopictus* abundance by calendar time in sites adjacent to directly treated areas

(**7**) Quantile of IIT-SIT intervention effectiveness to reduce *Ae. albopictus* abundance by time-since-intervention in sites adjacent to directly treated areas

(**8**) Quantile of IIT-SIT intervention effectiveness to reduce *Ae. albopictus* abundance by calendar time in sites adjacent to directly treated areas

(**9**) IIT-SIT intervention effectiveness to reduce *Ae. albopictus* abundance by time-since-intervention in core versus buffer areas

(**10)** IIT-SIT intervention effectiveness to reduce *Ae. albopictus* abundance by calendar time in sites in core versus buffer areas

The coloured lines represents the average values across the sectors. The shaded regions represent the pointwise 95% confidence intervals. The shaded regions in grey represent quantiles where there is an insignificant change in *Ae. albopictus* populations due to intervention. The shaded regions in other colours represent quantiles where there is a significant change in *Ae. albopictus* populations due to intervention. The arrows indicate whiskers truncated at the plot boundary.

**Fig D**

(1) Sector intervention effectiveness of IIT-SIT to reduce *Ae. albopictus* abundance over historical *Ae. albopictus* abundance quantiles in directly intervened sectors. Blue line represents GAM model fitting. Pseudo-log transformation applied in y-scale.

(2) Mean intervention effectiveness of IIT-SIT to reduce in *Ae. albopictus* abundance in direct intervened sectors by township. Coloured lines represent average IE across the sectors in each township. The shaded areas represent pointwise 95% confidence intervals.

**Robustness checks**

We conducted a large battery of robustness checks. Results are as follows:

- In-time placebo checks were done, with pseudo-treatment start dates for actual intervention sectors done 26 – 52 weeks prior to actual treatment (Fig E, I). Estimated intervention effectiveness on *Ae. aegypti* population was far smaller, with estimated median IEs of 1.14% and −0.53% for the pseudo-intervention period starting from 26 and 52 weeks prior to the start of the actual intervention respectively (Fig E1). Raw reductions in *Ae. aegypti* population in direct-intervened sectors between placebo quantile distribution and actual quantile distribution ranged from −0.442 – 0.162 and −0.377 – 0.189 for 26 and 52 weeks prior to the intervention, which were far smaller than the actual intervention effect.
- In-space placebo tests were conducted by removing the actual treated sectors, and repeating our analytical procedure by reassigning control sectors to be the pseudo-treated sector. We found that estimated raw reduction on each quantile of *Ae. aegypti* population was far smaller, and ranged from −0.008 – 0.017 for the pseudo-intervention control sectors (Fig F1).
- We repeated the in-time and in-space placebo checks for the endpoint of *Ae. albopictus* abundance and found that our analytical strategy did not reproduce significant intervention effectiveness estimates for the endpoint of *Ae. albopictus* abundance in pseudo-intervention sectors or time periods (Fig E3, F3).
- We repeated the in-time placebo checks for the spillover impact of *Ae. aegypti* and *Ae. albopictus* abundance and found that our analytical strategy did not reproduce significant intervention effectiveness estimates for the endpoint of *Ae. albopictus* abundance in pseudo-intervention time periods in spillover locations (Fig E4, E6, F4, F6).
- We conducted a visual inspection of counterfactual and actual quantile functions for every treated sector during its pre-treatment period to provide a direct comparison between DSC estimates and the actual situation (Fig G). The 95% confidence intervals of counterfactual quantile distributions were calculated by bootstrap procedure provided by Dijcke, Gunsilius and Wright. The resampling in bootstrap framework was repeated 500 times. We modified the calculation of resampled average weights by solving a plain sum optimization over the whole pre-treatment period rather than separately for each interval in pre-treatment period, thereby providing a joint estimation. Most of actual quantile functions fell into confidence intervals of counterfactual quantile function, indicating that DSC estimations closely fit actual situations. The result supports the reliability of counterfactual outcomes in post-treatment period.
- **Placebo permutation tests were conducted following Gunsilius. Post-intervention IEs were calculated for each control sector by removing the actual sector of interest, reassigning the control sector to be pseudo-treated sector, and repeating the analytical procedure. In each iteration, this produces an IE which is used to construct the permutation distribution. The p-value for the actual IE is taken to be the 1 minus the proportion of times the actual IE was less than the IEs in the permutation distribution**.

Result showed a statistically significant increase in the proportion of intervened sectors rejecting the null hypothesis that the treatment effect on intervened sectors does not result in a significant reduction on *Ae. Ae. aegypti* abundance at the 5% level (Fig H). Percentage of intervened sectors rejecting the null hypothesis of no significant reduction in relative *Ae. aegypti* abundance in IIT-SIT treated locations increased from 29.67% to 78.18% in the first intervention year and was maintained at around 90% for the rest of study period. This implied a significant treatment effect in directly intervened sectors. Similarly, the percentage of adjacent, non-treated sectors rejecting the null hypothesis of no significant reduction changed from 2.5% – 28.57% to around 50%. Two-sided hypothesis tests was applied to determine if changes in *Ae. albopictus* population due to *Wolbachia* were significant at the 5% level. The percentage of intervened sectors rejecting the null hypothesis of no statistically significant change in relative *Ae. albopictus* abundance in the first intervention year ranged between 23.08% – 45.31% in the first intervention year, and decreased to around 10% for the rest of study period, which implied a limited effect of *Wolbachia* on *Ae. albopictus* population over the trial period. A similar result on *Ae. albopictus* populations was found in spillover locations.

- We conducted a sensitivity analysis on the number of control sector used in the donor pool by varying the donor pool to comprise 40, 100 or 600 control sectors. We compared the 2-Wasserstein distances of counterfactual distributions constructed by different numbers of control sectors to the treated sector’s distributions during pre-treatment period to measure the estimators’ performance. We found no significant difference in performance when varying the number of control sectors (Fig J). Therefore, considering processing speed and computational burden, we selected the top 40 most correlated control sectors for every treated sector. The optimal weighting schemes of treated sectors shown that most of the control sectors were assigned zero weightage in constructing counterfactual quantile functions (Table A – G)

**Fig E**

1. In-time placebo test result on intervention efficacy in *Ae. aegypti* abundance in 26 and 52 weeks prior to actual treatment in direct treated sectors
2. In-time placebo test result on intervention efficacy in the proportion of traps which did not record mosquitoes in 26 and 52 weeks prior to actual treatment in direct treated sectors
3. In-time placebo test result on intervention efficacy in *Ae. albopictus* abundance in 26 and 52 weeks prior to actual treatment in direct treated sectors
4. In-time placebo test result on intervention efficacy in *Ae. aegypti* abundance in 26 and 52 weeks prior to actual treatment in sites adjacent to directly treated areas
5. In-time placebo test result on intervention efficacy in the proportion of traps which did not record mosquitoes in 26 and 52 weeks prior to actual treatment in sites adjacent to directly treated areas
6. In-time placebo test result on intervention efficacy in *Ae. albopictus* abundance in 26 and 52 weeks prior to actual treatment in sites adjacent to directly treated areas

**Fig F**

1. In-space placebo test results on raw increment of proportion of traps which did not record mosquitoes by time-since-intervention in directly intervened sites in *Ae. aegypti* abundance study
2. In-space placebo test result on average raw reduction of *Ae. aegypti* abundance by time-since-intervention in directly intervened sites in *Ae. aegypti* abundance study
3. In-space placebo test result on average raw reduction of *Ae. albopictus* abundance by time-since-intervention in directly intervened sites in *Ae. albopictus* abundance study
4. In-space placebo test results on raw increment of proportion of traps which did not record mosquitoes by time-since-intervention in sites adjacent to directly treated areas in *Ae. aegypti* abundance study
5. In-space placebo test result on average raw reduction of *Ae. aegypti* abundance by time-since-intervention in sites adjacent to directly treated areas in *Ae. aegypti* abundance study
6. In-space placebo test result on average raw reduction of *Ae. albopictus* abundance by time-since-intervention in sites adjacent to directly treated areas in *Ae. albopictus* abundance study

**Fig G**

*Ae. aegypti* abundance quantile distributions for intervened sectors in pre-intervention period and their respective counterfactuals as estimated using distributional synthetic controls. Shaded area represents pointwise 95% confidence intervals of the counterfactual quantile distribution.

**Fig H**

*P*-value plots for the placebo permutation test in studying the impact of *Wolbachia* IIT-SIT on *Ae.* *aegypti* abundance. The null hypothesis for the permutation test is that there is no effect of *Wolbachia* IIT-SIT on *Ae. aegypti* abundance in the specific post-intervention time interval. Blue solid line represents significance level $\alpha$ = 0.05. Green dash line represents the first epidemiological week of treatment.

**Fig I**

Visualization of in-time placebo test in the pseudo-intervention period across each intervened sector. Lines in the legend represents the time interval before the actual post intervention period (1 – 13 week, 14 – 26 week, 27 – 39 week and 40 – 52 week) and the pseudo-intervention period (26 weeks, 52 weeks).

**Fig J** 2-Wasserstein distance between actual distribution and counterfactual distributions constructed using different donor pool sizes. Numbers on the X-axis represent the last week in the 13-week interval in pre-treatment period, e.g. “−1” means “1 – 13 weeks before intervention”

**Table A**

Donor weights for the 9 Bukit Batok sectors (total 9) in studying *Ae. aegypti* abundance. Only sectors with any contribution to construct counterfactual distribution for any intervened sector are listed. A darker background colour represents a higher weight.

**Table B**

Donor weight for 15 Choa Chu Kang sectors (total 15) in studying *Ae. aegypti* abundance. Only sectors with any contribution to the counterfactual distribution for any intervened sector are listed. A darker background colour represents a higher weight.

**Table C**

Donor weight for Tampines sectors (total 38) in studying *Ae.* *aegypti* abundance. Only sectors with any contribution to construct counterfactual distribution for any intervened sector are listed. A darker background colour represents a higher weight.

**Table D**

Donor weight for Tampines sectors (total 38) in studying *Ae.* *aegypti* abundance. Only sectors with any contribution to the counterfactual distribution for any intervened sector are listed. A darker background colour represents a higher weight.

**Table E**

Donor weight for Tampines sectors (total 38) in studying *Ae.* *aegypti* abundance. Only sectors with any contribution to the counterfactual distribution for any intervened sector are listed. A darker background colour represents a higher weight.

**Table F**

Donor weight for 1^st^ – 15^th^ Yishun sector (total 29) in studying *Ae. aegypti* abundance. Only sectors with any contribution to construct counterfactual distribution for any intervened sector are listed. A darker background colour represents a higher weight.

**Table G**

Donor weight for Yishun sectors (total 29) in studying *Ae.* *aegypti* abundance. Only sectors with any contribution to the counterfactual distribution for any intervened sector are listed. A darker background colour represents a higher weight.

**Fig K**

Intervention effectiveness of IIT-SIT on *Ae.* *aegypti* abundance by sector. Columns represent the respective time periods of EW27 – 53 2020, EW1 – 26 2021, EW27 – 52 2021 and EW1 – 26 2022. Rows represent each township. From top to bottom: Bukit Batok, Choa Chu Kang, Tampines and Yishun townships are represented. Base layer from the Master Plan 2019 Land Use Layer dataset, licensed under the Singapore Open Data Licence version 1.0.

**Fig L**

Intervention effectiveness of IIT-SIT on *Ae.* *aegypti* abundance by sector. Columns represent the respective intervention time periods of 1 – 26, 27 – 52, 53 – 78, 79 – 104 and 105 – 124 weeks after intervention. Rows represent each township. From top to bottom: Bukit Batok, Choa Chu Kang, Tampines and Yishun townships are represented. Base layer from the Master Plan 2019 Land Use Layer dataset, licensed under the Singapore Open Data Licence version 1.0.

**Fig M**

Spillover effectiveness of IIT-SIT on *Ae.* *aegypti* abundance by sector. Columns represent the respective time periods of EW27 – 53 2020, EW1 – 26 2021, EW27 – 52 2021 and EW1 – 26 2022. Rows represent source township of spillover effect. From top to bottom: combination of Bukit Batok and Choa Chu Kang, Yishun and Tampines. Blue outlier sectors highlight intervened sector during each period. Base layer from the Master Plan 2019 Land Use Layer dataset, licensed under the Singapore Open Data Licence version 1.0.

**Fig N**

Spillover effectiveness of IIT-SIT on *Ae.* *aegypti* abundance by sector. Columns represent the respective time periods of 1 – 26, 27 – 52, 53 – 78, 79 – 104 and 105 – 124 weeks after intervention. Rows represent source township of spillover effect. From top to bottom: combination of Bukit Batok and Choa Chu Kang, Yishun and Tampines. Base layer from the Master Plan 2019 Land Use Layer dataset, licensed under the Singapore Open Data Licence version 1.0.

**Fig O**

Intervention effectiveness of IIT-SIT on *Ae.* *albopictus* abundance by sector. Columns represent the respective time periods of EW27 – 53 2020, EW1 – 26 2021, EW27 – 52 2021 and EW1 – 26 2022. Rows represent each township. From top to bottom: Bukit Batok, Choa Chu Kang, Tampines and Yishun townships are represented. Base layer from the Master Plan 2019 Land Use Layer dataset, licensed under the Singapore Open Data Licence version 1.0.

**Fig P**

Intervention effectiveness of IIT-SIT on *Ae.* *albopictus* abundance by sector. Columns represent the respective intervention time periods of 1 – 26, 27 – 52, 53 – 78, 79 – 104 and 105 – 124 weeks after intervention. Rows represent each township. From top to bottom: Bukit Batok, Choa Chu Kang, Tampines and Yishun townships are represented. Base layer from the Master Plan 2019 Land Use Layer dataset, licensed under the Singapore Open Data Licence version 1.0.

**Fig Q**

Spillover effectiveness of IIT-SIT on *Ae.* *albopictus* abundance by sector. Columns represent the respective time periods of EW27 – 53 2020, EW1 – 26 2021, EW27 – 52 2021 and EW1 – 26 2022. Rows represent source township of spillover effect. From top to bottom: combination of Bukit Batok and Choa Chu Kang, Yishun and Tampines. Blue outlier sectors highlight intervened sector during each period. Base layer from the Master Plan 2019 Land Use Layer dataset, licensed under the Singapore Open Data Licence version 1.0.

**Fig R**

Spillover effectiveness of IIT-SIT on *Ae.* *albopictus* abundance by sector. Columns represent the respective time periods of 1 – 26, 27 – 52, 53 – 78, 79 – 104 and 105 – 124 weeks after intervention. Rows represent source township of spillover effect. From top to bottom: combination of Bukit Batok and Choa Chu Kang, Yishun and Tampines. Base layer from the Master Plan 2019 Land Use Layer dataset, licensed under the Singapore Open Data Licence version 1.0.

**Fig S**

Raw increase on percentages of traps with zero wild *Ae. aegypti* mosquito by sector. Columns represent the respective time periods of EW27 – 53 2020, EW1 – 26 2021, EW27 – 52 2021 and EW1 – 26 2022. Rows represent each township. From top to bottom: Bukit Batok, Choa Chu Kang, Tampines and Yishun townships are represented.

Base layer from the Master Plan 2019 Land Use Layer dataset, licensed under the Singapore Open Data Licence version 1.0.

**Sensitivity analysis**

We conducted a large battery of sensitivity analysis. Results are as follows:

(**A**) We conducted a sensitivity check on different choices of distance metrics in weighting optimization. Jensen-Shannon divergence (JS divergence) is a method to measure the differences between two probability distribution. 1-Wasserstein distance is a method to measure the differences of cumulative mass functions for discrete and ordinal variables. Our targeted variable is the scenario of mosquito elimination in a sector. Two possible values for the discrete variable are “Eliminated” and “Not eliminated”. The probability distribution for this variable is a Bernoulli distribution. Therefore, both 1-Wasserstein distance and JS divergence are possible distance metrics in weighting optimization. We separately ran weighting optimization for these two methods and compared the counterfactual outcome differences to the actual quantile functions for the whole pre-treatment period for each treated sector. The results are quantified via the 1-Wasserstein distance. A larger difference represents poorer model fit in the pre-intervention period. Counterfactual outcomes using 1-Wasserstein distance fitted the observed distributions better (See Fig T). 1-Wasserstein distance was therefore chosen as the distance metric in weighting optimization for this outcome variable.

(**B**) We conducted a sensitivity analysis check on the choice of sampling distributions in the weight optimization step. We considered the uniform distribution and Beta (3,3) distribution to randomly select quantiles. We graphically visualized the difference in outcomes in the post-intervention period using either sampling distribution to examine the influence of the sampling distribution on our results. There were no major differences in results when employing either sampling distribution (Fig U).

**Fig T**

Values of differences between counterfactual CDF and actual CDF during pre-intervention period using the two different distance metrics used to optimize weights for the distributional synthetic controls in studying *Ae. aegypti* elimination. Red line represents the difference between actual CDF and counterfactual CDF calculated by optimal weight based on 1-Wasserstein distance. Blue line represents the difference between actual CDF and counterfactual CDF calculated by optimal weight based on Jensen-Shannon divergence. A larger difference represents poorer model fit in the pre-intervention period.

**Fig U**

Difference in values between counterfactual quantile functions computed via sampling using the beta or uniform distribution in studying *Ae. aegypti* abundance. Red line represents the raw GAI difference between actual and counterfactual quantile distributions calculated from Beta(3,3) sampling distribution. The blue line represents the raw GAI difference between actual and counterfactual quantile functions calculated from uniform sampling distribution. Each line represents one pre-treatment time interval in the pre-treatment period.
