# Supplementary material for: Heterogeneous suppressive effect of Wolbachia incompatible insect technique coupled with sterile insect technique across time and historical Ae. aegypti abundance - using distributional synthetic controls
Source: PLoS Comput Biol. 2026 Jun 22;22(6):e1014355. doi: 10.1371/journal.pcbi.1014355 (PMC13336471; doi:10.1371/journal.pcbi.1014355)
Supplement: Attachment — Submitted filename: PLoS_CB_report.pdf [file pcbi.1014355.s002.pdf]

Report on
"Heterogenous suppressive effect of Wolbachia incompatible insect technique coupled with sterile insect technique across time and historical *Ae. aegypti* abundance - using distributional synthetic controls"

Summary

This paper applies distributional synthetic control (DSC) methods to evaluate the effectiveness of Wolbachia-based incompatible insect technique coupled with sterile insect technique (IIT-SIT) for suppressing *Aedes aegypti* populations in Singapore. The authors leverage trap-level mosquito abundance data from a large-scale field trial to quantify heterogeneous treatment effects across time, historical abundance levels, and spatial configurations (direct treatment vs. spillover areas). The key findings are that intervention effectiveness (IE) increases over time, reaching up to 86% suppression, with substantial spillover effects in adjacent untreated areas.

The paper addresses an important public health question using a novel methodological approach. However, I have several concerns regarding both the methodological implementation and the identification strategy that should be addressed before publication.

Major Comments

1. Justification for Smoothing the Quantile Functions
The DSC method of Gunsilius (2023) applies to general distributions, including discrete empirical CDFs, without requiring smoothing. The authors smooth the quantile functions using linear interpolation (line 125) but provide no formal justification for this choice. I would like to see either:

   (a) A rigorous argument for why smoothing is necessary or beneficial in this application, including any implications for the theoretical properties of the estimator; or
   (b) Results using the raw empirical distribution without smoothing, to assess sensitivity of the findings to this choice.

   Note that for the binary elimination outcome, the authors appropriately use the 1-Wasserstein distance on the discrete CDF, which is consistent with the DSC framework.

2. Definition of the "Perfect" Quantile Function
The authors define $F^{-1}_{\text{perfect}}(q) = 0$ for all $q \in [0, 1]$ (line 224-225). However, this is not a valid quantile function. A proper quantile function must be non-decreasing and right-continuous, with $\lim_{q \to 1} F^{-1}(q) = \sup\{x : F(x) < 1\}$.

   I believe the authors intend to represent a degenerate distribution concentrated at zero, for which the quantile function is:

$$F^{-1}_{\text{perfect}}(q) = 0 \quad \text{for all } q \in (0, 1],$$

with the understanding that this corresponds to the CDF $F_{\text{perfect}}(x) = \mathbf{1}\{x \geq 0\}$. The authors should clarify this point and ensure their numerical implementation correctly handles this degenerate case when computing Wasserstein distances.

3. Justification for the Intervention Effectiveness Measure
The intervention effectiveness measure is defined as (lines 222-223):

$$IE_{d,t} = \frac{\widehat{W}_2(F^{-1}_{cf,d,t}, F^{-1}_{\text{perfect}}) - \widehat{W}_2(F^{-1}_{d,t}, F^{-1}_{\text{perfect}})}{\widehat{W}_2(F^{-1}_{cf,d,t}, F^{-1}_{\text{perfect}})}.$$

While this has an intuitive interpretation as a relative reduction in "distance to elimination," the choice requires more justification:

- Why use distance to a perfect (zero mosquito) benchmark rather than a direct comparison between treated and counterfactual distributions?
- How does this measure relate to more standard treatment effect measures in the DSC literature?
- The measure can exceed 100% or become negative in certain configurations. What is the interpretation in these cases?

A more detailed discussion connecting this measure to the causal estimand of interest would strengthen the paper.

4. Selection into Treatment Timing
The paper does not adequately address how sectors were selected for treatment and in what order. If treatment was prioritized based on mosquito abundance, dengue risk, or other factors correlated with potential outcomes, this creates *confounding* that synthetic controls alone may not fully address.

Specifically, the finding that sectors with lower historical GAI exhibit higher IE (Figure 4A) could reflect:

(a) A true moderating effect of baseline abundance; or
(b) Selection bias if high-abundance areas were treated first and these areas have systematically different trajectories.

The authors should clearly describe the treatment assignment mechanism and discuss how their identification strategy addresses potential selection on observables and unobservables (case (b)). In particular, is it possible to check if the trajectories are systematically different?

5. Pre-Screening of Donor Pool
The procedure of selecting the top 40 most correlated control sectors before optimization (lines 144-151) is not part of the original Gunsilius (2023) DSC methodology. This data-dependent pre-selection step needs to be argued better. In particular, the results might be sensitive to the choice of 40 at the cutoff. The authors should present sensitivity analyses varying the number of pre-selected controls.

6. Spillover Contamination of the Donor Pool
The authors acknowledge that control sites not adjacent to treatment areas may still experience interference (lines 378-380), but dismiss this concern by citing limited dispersal of *Ae.*

aegypti. However, the paper's own results show spillover effects reaching 78% in adjacent areas. This creates a logical tension in my opinion: if Wolbachia-infected mosquitoes disperse sufficiently to suppress wild-type populations in adjacent sectors, they may also reach "control" sectors that are merely non-adjacent. Contamination of the donor pool would bias IE estimates downward, but the magnitude is unknown. I want the authors to address this briefly.

Minor Comments

1. **Inference procedure** The bootstrap procedure (lines 244-247) resamples trap-level observations within sectors, which captures sampling variability and is perfectly fine. Reading the authors text, it seems like they conflate the bootstrap estimation (which exclusively deals with the randomness of the sampling), and the permutation test (which deals with the "causal interpretation"). The authors should be clearer at distinguishing these. Both are needed as they "test" different things.

2. **Beta(3,3) sampling:** The modification replacing uniform sampling with Beta(3,3) sampling (lines 158-161) is inadequately justified. Why this specific distribution? How sensitive are results to this choice?

3. **Negative spillover IE:** The reported spillover IE of -1.29% in weeks 1–52 (line 292) deserves explanation. Does this suggest the intervention temporarily increased mosquito abundance in adjacent areas, perhaps through displacement? Or is this an artifact of estimation error? The biological interpretation matters.

4. **Core vs. buffer distinction:** The distinction between core sectors (no border with controls) and buffer sectors (border with controls) is introduced in lines 203-205 but not systematically exploited in the results. Given the paper's focus on spatial heterogeneity, separate analyses for these categories would be informative.

5. **Writing quality:** The manuscript contains numerous grammatical errors and typos that impede readability. Examples include:

   - "Heterogenous" should be "Heterogeneous" throughout.
   - Line 54: "complements environmental management (REF)" – missing reference.
   - Line 189: "deviatesfrom" should be "deviates from."
   - Various instances of inconsistent capitalization and punctuation.
