# Supplementary material for: Heterogeneous suppressive effect of Wolbachia incompatible insect technique coupled with sterile insect technique across time and historical Ae. aegypti abundance - using distributional synthetic controls
Source: PLoS Comput Biol. 2026 Jun 22;22(6):e1014355. doi: 10.1371/journal.pcbi.1014355 (PMC13336471; doi:10.1371/journal.pcbi.1014355)
Supplement: Attachment — Submitted filename: Wolbachia2.pdf [file pcbi.1014355.s005.pdf]

Report on Review manuscript
Manuscript: PCOMPBIOL-D-25-02450R1

The authors have done a thorough job addressing the concerns raised in the first round. The added sensitivity analyses (donor pool size, sampling distribution), the clarification of the IE measure and degenerate distribution, the bootstrap/permutation distinction, and the core-versus-buffer comparison are all satisfactory. I have only two minor remaining points before I can recommend acceptance.

1. **Treatment assignment mechanism (previously Major Comment 4).** The response letter clearly explains that Yishun and Tampines were selected for an expanding release strategy, while Bukit Batok and Choa Chu Kang were selected for targeted releases in areas with high *Ae. aegypti* abundance and persistent dengue transmission. However, this information does not appear in the manuscript itself. I ask the authors to add two to three sentences in the Methods section (around lines 97–119) describing the rationale for township selection and the different release strategies employed, citing Bansal et al. (Ref. 4) as appropriate. This context is important for readers who will not have access to the response letter.

2. **Abstract inconsistency and residual typos (previously Minor Comment 5).** The abstract (line 24) states that traps were "routinely maintained and measured on a fortnight basis," but the revised Methods section (line 103) now states that each trap was "measured twice a week." These are contradictory and the abstract must be corrected. Additionally, a few typos remain in the clean manuscript:

    - Line 107: "Similar" → "Similarly"
    - Line 242: "so that is can be scored" → "so that it can be scored"
    - Line 242: "allowed us to calculating" → "allowed us to calculate"

    I ask the authors to perform a careful final proofread before resubmission.
