# Supplementary material for: Heterogeneous suppressive effect of Wolbachia incompatible insect technique coupled with sterile insect technique across time and historical Ae. aegypti abundance - using distributional synthetic controls
Source: PLoS Comput Biol. 2026 Jun 22;22(6):e1014355. doi: 10.1371/journal.pcbi.1014355 (PMC13336471; doi:10.1371/journal.pcbi.1014355)
Supplement: Attachment — Submitted filename: Reviewer report.docx [file pcbi.1014355.s003.docx]

**REVIEWER REPORT**

The authors proposed a new method of causal inference to estimate the efficacy of Wolbachia in suppressing *Ae. aegypti* abundance. This new method attempted to characterise effectiveness across the distribution of values rather than just looking at the effect on the average, accounted for smaller-scale spatial heterogeneity in the effects, and also examined the drivers of these heterogeneities (i.e., adjacency and proximity to untreated areas). This study involves high-resolution spatiotemporal data from Wolbachia trials in Singapore, with a synthetic control method used to estimate effectiveness.

Overall, it is an interesting study and method developed, but I have several comments that I hope the authors can clarify to make the manuscript clearer.

**Major comments**

**Species-specific elimination definition**

No elimination = no *Aedes aegypti*/*albopictus*? Why is *Aedes albopictus* considered here, while the released mosquitoes are *Aedes aegypti*, and the objective is to measure the impact on *Aedes aegypti*? Would removing the *Aedes albopictus* count increase the impact estimate? Surely the probability number shows a very low probability of elimination, but shouldn't the calculation consider *Aedes aegypti* only to make the elimination probability calculation exclusive for *Aedes aegypti* (since the trial released *Aedes aegypti* only)?

**Trial protocol**

What is not clear here is how many times the mosquitoes were released during the observation period? What is the density of the release? Are they all the same for each sector? Any frequency and periodicity that may affect the observations and thus the analysis? Was it done one time at the beginning? And nothing was done regarding the release during the observation period? I understand that previous studies have discussed this, but it would help readers unfamiliar with this trial by providing guidance on the trial's designs, thereby helping them connect it more easily to the results.

**Data interpolation methods for odd weeks**

Data were collected every two weeks, but how do you generate data for odd weeks (i.e., observation weeks start from week 1 and end in week 13)? How does the algorithm calculate the data to account for week 13, as, from my understanding, observations must be made at week 14?

**Synthetic control site reuse and township boundaries**

Can a control from the donor pool be used multiple times for different intervention sites if they have high correlations for those sites relative to others? Or can each control only be used for a single intervention site? If they can be used multiple times, are there instances in which a single site from the donor pool served as a synthetic control across multiple intervention sites, thereby driving the results? Or was the weighting different for this site across those intervention sites?

Can a site in a different township serve as a control site? If yes, will this affect the results?

**Pseudo-intervention definition**

What does assigning a pseudo-intervention mean? Does that mean just assigning them as intervention sites (without actually changing any values at those sites), and hence the hypothesis is that there will be no difference? Or some intervention efficacy assigned to these sites (with known values) to test the hypothesis?

**Table 1 discrepancy**

In Table 1, why are the numbers for Direct not equal to the sum of Core and Buffer? Isn't the Direct one supposed to mean accounting for both based on the definition?

**Seasonal mosquito abundance heterogeneity and adjustment**

Is there any seasonal heterogeneity in mosquito abundance across the study sites? If yes, how did the IE calculation account for this? In the method, it was noted that calendar time and event time adjustments were performed. But it is not clear whether there's seasonality in mosquito abundance, whether the adjustment accounts for it, and whether the IE is, importantly, not biased by seasonality at the sites that adopted treatment later in the study.

**Core versus Buffer differentiation**

I feel more elaboration is needed in the Core and Buffer areas' IE. From my understanding, it is shown in Figure 3. However, there's little elaboration on whether there's any difference in IE between those places, even though the study was designed to detect it. In general, figure results were not elaborated clearly and completely and some of them were left to readers’ interpretation. I suggest the authors to provide more elaborations on the figures shown in the manuscript to guide interpretations.

**Comparison with prior studies**

I think in the discussion, it may help to provide context for the estimates of IE from previous studies using similar data (with a different approach). Are there any differences in estimates when understanding the distributional IE rather than just the average, and when accounting for smaller-scale spatial-level data? Are the overall estimates ultimately the same (i.e., township-level estimates)?

**Future methodological and trial design guidance**

I think the discussions could also be improved by relating the results to other study contexts and by outlining what to develop further. Since the method is also novel, can the authors suggest how data collection or study design could be improved to better apply the DSC method in the future (e.g., so less distributional adjustment is needed, and trial designs will then support the establishment of this type of analysis)? Hence, can the method then be applied with fewer assumptions and fewer smoothing steps?

**Minor points**

- Abstract, Line 38: GAI hasn't been defined
- Line 54: Not sure if (REF) there refers to a planned reference to be added or an acronym for previous words
- Line 356: "Conventional" came up twice
- Terminology: For consistency, write mosquito species as *Ae. aegypti* throughout rather than just "aegypti"
- Figure 2: Suggest that the scale for the y-axis in panels C and D should be made similar for ease of comparison
