# Supplementary material for: Heterogeneous suppressive effect of Wolbachia incompatible insect technique coupled with sterile insect technique across time and historical Ae. aegypti abundance - using distributional synthetic controls
Source: PLoS Comput Biol. 2026 Jun 22;22(6):e1014355. doi: 10.1371/journal.pcbi.1014355 (PMC13336471; doi:10.1371/journal.pcbi.1014355)
Supplement: Attachment — Submitted filename: Full_response_Feb11.docx [file pcbi.1014355.s004.docx]

**Editorial Letter**

Dear Dr. Zhai,

Thank you for submitting your manuscript to PLOS Computational Biology. After careful consideration, we feel that it has merit but does not fully meet PLOS Computational Biology's publication criteria as it currently stands. Therefore, we invite you to submit a revised version of the manuscript that addresses the points raised during the review process. Reviewer 2 has raised concerns about the novelty of the work given similar findings have been published in a 2024 paper. When responding to the reviewer the authors should consider whether edits to the manuscript should also be made to ensure the novelty of this work is clear to all readers. In addition to the reviewer's comments, I think the results section is currently quite brief --- if you could include more of the elaboration and interpretation of results it would likely help readers follow your findings (you could also more explicitly guide the readers to the specific results in the supplementary).

Please submit your revised manuscript by Mar 08 2026 11:59PM. If you will need more time than this to complete your revisions, please reply to this message or contact the journal office at ploscompbiol@plos.org. When you're ready to submit your revision, log on to https://www.editorialmanager.com/pcompbiol/ and select the 'Submissions Needing Revision' folder to locate your manuscript file.

Please include the following items when submitting your revised manuscript:

* A letter that responds to each point raised by the editor and reviewer(s). You should upload this letter as a separate file labeled 'Response to Reviewers'. This file does not need to include responses to formatting updates and technical items listed in the 'Journal Requirements' section below.
* A marked-up copy of your manuscript that highlights changes made to the original version. You should upload this as a separate file labeled 'Revised Manuscript with Track Changes'.
* An unmarked version of your revised paper without tracked changes. You should upload this as a separate file labeled 'Manuscript'.

If you would like to make changes to your financial disclosure, competing interests statement, or data availability statement, please make these updates within the submission form at the time of resubmission. Guidelines for resubmitting your figure files are available below the reviewer comments at the end of this letter

We look forward to receiving your revised manuscript.

Kind regards,

Oliver Eales
Academic Editor
PLOS Computational Biology

Roger Kouyos
Section Editor
PLOS Computational Biology

**Response We thank the editor for the opportunity to revise our manuscript entitled “***Heterogeneous suppressive effect of Wolbachia incompatible insect technique coupled with sterile insect technique across time and historical Ae. aegypti abundance - using distributional synthetic controls***”. We hope that the revised manuscript below provides clarity and cogency to our study, and adheres to the requirements of the journal.**

**We have now augmented the discussion section to clarify the novelty of DSC compared to the SCM approach in previous work:**

*“IE estimates yielded by DSC indicated treatment effect heterogeneity on both spatial and temporal scales. In contrast, the canonical SCM yielded pointwise IE estimates and showed temporal heterogeneity of treatment effects. In the canonical SCM, the intervention effectiveness of Wolbachia is evaluated via average mosquito abundance in sectors over time, however, this disregards the finer-scale trap-level information in sectors. Whereas DSC yields intervention effectiveness estimates using the entire quantile of observed mosquito abundance within sectors which contains more information about variation and spread of data in those evaluated sectors. By obtaining counterfactual quantile functions as the comparator group to the observed quantile function in intervention sites, IE estimates can be expressed in other statistics, such as the IE over different mosquito abundance quantiles, and help tease out heterogeneous treatment effects over smaller-scale spatial scales.”*

**We have now described key findings from the results section to help readers understand this treatment effect heterogeneity. Additional explanations and graphs were linked to the supplementary information section:**

1. **We added descriptions of changes in IE over different mosquito abundance quantiles, explained common features of these trajectories and compared IE results between different rankings shown as quantiles.**

***“By comparing the IEs over different quantiles, we found that in directly treated sectors, quantile IEs around the 25^th^ quantile were higher than that at other quantiles. Quantile IEs around the 0^th^ quantile were lower and gradually increased as quantiles increased. Comparing quantile IE between the 25^th^ – 49.9^th^ quantile and 75^th^ – 100^th^ quantile , the maximum quantile IE difference decreased from 61.33% in 1 – 13 weeks after intervention to 29.62% in week 105 – 117, and the minimum quantile IE difference decreased from 24.40% to −0.7%. This showed that IE reduced quickly at higher GAI quantiles at the beginning of treatment, and treatment effect was lower in moderate mosquito abundance areas in the sector. Longer treatment times could however shrink the difference on treatment effectiveness between mosquito quantiles…”***

**2. We explained spatial heterogeneity of estimated IEs. We also linked the explanation to our supplementary information (SI) material.**

***“We further studied possible treatment effect heterogeneity across different spatial locations in intervened sectors. By comparing IEs over intervention time between buffer and core sectors, we found similar trajectories in treatment effect. During the first 52-weeks of treatment, average IE in core areas increased from 28.09% (95%CI: 17.78%–40.81%) to 62.63% (95%CI: 48.41%–81.78%), while average IE in buffer areas increased from 20.71% (95%CI: 11.52%–29.65%). However, we found no statistically significant differences in IEs between core and buffer sectors over time (Welch’s T-test pval<0.05).***

***IE in both buffer and core sectors increased over the study period. However, due to the staggered adoption of treatment, sectors in buffer zones received longer treatment times compared to those in core zones and therefore stronger treatment effects in the same calendar time.”***

**3. We explained the negative IE value estimated and presented in the spillover IE graph.**

“… *Raw reductions in abundance were also greater in spillover locations that were subject to longer intervention periods (Figure 2D). The result indicated an insignificant but negative intervention effectiveness estimate in sites defined to be spillover locations at the start of the trial. This potentially reflects sampling noise and migration of wild-type mosquitoes at earlier treatment times.”*

**4. We elaborated on our findings on treatment effect heterogeneity being driven by historical abundance as well:**

***“…Intervention effectiveness increased with more intervention time with little heterogeneity across intervened townships (Figure 4B). Areas with historical high mosquito populations required 52 weeks to reach 75% suppression while areas with low historical mosquito populations required 26 weeks.”***

**Additional Editor Comments (if provided):**

**Journal Requirements:**

**If the reviewer comments include a recommendation to cite specific previously published works, please review and evaluate these publications to determine whether they are relevant and should be cited. There is no requirement to cite these works unless the editor has indicated otherwise.**

**Reviewer #1 comments**:

**Major Comment 1:** Justification for Smoothing the Quantile Functions

The DSC method of Gunsilius (2023) applies to general distributions, including discrete empirical CDFs, without requiring smoothing. The authors smooth the quantile functions using linear interpolation (line 125) but provide no formal justification for this choice. I would like to see either: (a) A rigorous argument for why smoothing is necessary or beneficial in this application, including any implications for the theoretical properties of the estimator; or (b) Results using the raw empirical distribution without smoothing, to assess sensitivity of the findings to this choice.

Note that for the binary elimination outcome, the authors appropriately use the 1-Wasserstein distance on the discrete CDF, which is consistent with the DSC framework.

**Response: We thank the reviewer for the comment on smoothing quantile functions. The smoothing was applied only to construct the continuous empirical quantile distribution. In this study, we calculated the average GAI (Gravitrap *Ae. aegypti* index) per postal code per week in the sector area over a time interval. The average GAI was a positive continuous variable bounded by 0. The quantile function aims to reflect the probability distribution of GAI values averaged over each postal code and each week. For computation convenience, the 2-Wasserstein distance calculation in this study is converted from an integral to a summation (Line 187).**

**As the average GAI value by postal code is a continuous variable, smoothing the empirical quantile functions is an appropriate approach to fit the actual quantile function rather than the direct inverse of cumulative density function, which never consider the continuous nature of the variable. This is also the default implementation scheme for treating continuous variables in the DSC framework. Please see the DiSCos package documentation for more details**^1^**. We have revised our methods section for clarity:**

***“****These observations were then used to construct continuous, quarterly, sector-level empirical quantile distributions* $F^{-1}$ *of average trap-level number of trapped wild female Ae. aegypti mosquitoes for a specific postal code in that sector, which were subsequently smoothened using linear interpolation. The default algorithm of empirical quantile functions in DSC framework was implemented for treating continuous variables.* ***”***

**Major Comment 2:** Definition of the “Perfect” Quantile Function

The authors define $F_{perfect}^{-1}\left( q \right)=0$ for all $q\in[0,1]$ (line 224-225). However, this is not a valid quantile function. A proper quantile function must be non-decreasing and right-continuous, with $\lim_{q\to1} F^{-1}\left( q \right)=sup\{x:F\left( x \right)<1\}$. I believe the authors intend to represent a degenerate distribution concentrated at zero, for which the quantile function is: $F_{perfect}^{-1}\left( q \right)=0$ for all $q\in(0,1]$, with the understanding that this corresponds to the CDF $F_{perfect}\left( x \right)=\boldsymbol{1}\{x\geq0\}$. The authors should clarify this point and ensure their numerical implementation correctly handles this degenerate case when computing Wasserstein distances.

**Response:** We thank the reviewer for the comment and agree with the reviewer. $F_{perfect}^{-1}\left( q \right)=0$ is not a valid quantile function. However, the degenerate distribution is used for calculating the 2-Wasserstein distance with other quantile functions, and the 2-Wasserstein distance only requires distributions with finite second moment. $F_{perfect}^{-1}\left( q \right)=0$ has a finite second moment $E\left( X^{2} \right)=0$. Therefore, the degenerate distribution can be used in 2-Wasserstein distance calculation. We have revised our methods section for clarity:

*“For a specific sector d, the IE at time t was calculated as follow:*

$$IE_{d,t}= \frac{\hat{W_{2}}\left( F_{cf,d,t}^{-1}, F_{perfect}^{-1} \right)- \hat{W_{2}}(F_{d,t}^{-1}, F_{perfect}^{-1})}{\hat{W_{2}}(F_{cf,d,t}^{-1}, F_{perfect}^{-1})}$$

*where* ${F_{perfect}}^{-1}$ *is a degenerate distribution concentrated at zero with* ${F_{perfect}}^{-1}(q)=0, q \epsilon\left[ 0,1 \right]$ *and characterizes the scenario with zero mosquitoes in the sector.”*

**Major Comment 3:** Justification for the Intervention Effectiveness Measure

The intervention effectiveness (IE) measure is defined as (lines 222-223): $IE_{d,t}= \frac{\hat{W_{2}}\left( F_{cf,d,t}^{-1}, F_{perfect}^{-1} \right)- \hat{W_{2}}(F_{d,t}^{-1}, F_{perfect}^{-1})}{\hat{W_{2}}(F_{cf,d,t}^{-1}, F_{perfect}^{-1})}$. While this has an intuitive interpretation as a relative reduction in “distance to elimination”, the choice requires more justification:

- Why use distance to a perfect (zero mosquito) benchmark rather than a direct comparison between treated and counterfactual distributions?
- How does this measure relate to more standard treatment effect measures in the DSC literature?
- The measure can exceed 100% or become negative in certain configurations. What is the interpretation in these cases?

A more detailed discussion connecting this measure to the causal estimand of interest would strengthen the paper.

**Response: We thank the reviewer for the comments.**

(1) **Current DSC literature does not provide a standard formula to determine treatment effects. In practice, raw quantile value differences were taken to be treatment effects**^2,3^**. In the results section, we presented raw treatment effects (See Figure 2C below):**

***Figure 2C Raw reduction distribution in Ae. aegypti abundance by time-since-intervention in directly intervened sites***

(2) **However, this measurement of IE does not reflect the relative treatment effect of a treated unit. To elaborate, a relative measure would be as the following:** $\tilde{IE}_{d,t}= \frac{f\left( F_{cf,d,t}^{-1},F_{d,t}^{-1} \right)}{f(F_{cf,d,t}^{-1})}\times100$**, but the numerator quantity would be a difference between two quantiles (i.e a 2-Wasserstein distance), whereas the denominator would be the quantile itself, which is on a different scale.**

**Therefore, we introduced a perfect (zero mosquito) baseline as the distribution to be compared against. The score of each distribution is defined as the 2-Wasserstein distance between its quantile distribution and the perfect baseline. The difference of two quantile distributions (the observed quantile and the counterfactual quantile) are interpreted as the difference of distributions’ scores. We have now further elaborated on our reasoning:**

*“Current DSC literature only proposed quantifying treatment effects via raw quantile value differences. This represented raw rather than relative treatment effects. We introduced a baseline scenario of no mosquitoes being detected, so that is can be scored against the mosquito abundance for a sector. This allowed us to calculating 2-Wasserstein distances between the GAI distribution at the sector and the baseline scenario. To compute IEs, we took the differences in scores of the intervened location’s mosquito abundance…”*

(3) **The measure** $IE_{d,t}= \frac{\hat{W_{2}}\left( F_{cf,d,t}^{-1}, F_{perfect}^{-1} \right)- \hat{W_{2}}(F_{d,t}^{-1}, F_{perfect}^{-1})}{\hat{W_{2}}(F_{cf,d,t}^{-1}, F_{perfect}^{-1})}$ **cannot exceed 100%, as the 2-Wasserstein distance cannot produce a non-negative value. The maximum measure will be 1 and occur only if the actual distribution reflects a scenario where mosquitoes are eliminated in the treated sector. However, the IE measure can be negative if the intervention effectiveness is negative. This would indicate that the actual mosquito abundance distribution is farther from the scenario of mosquito elimination versus the counterfactual outcome in the intervened sector. We have now clarified this in the text:**

***“…****The IE has a range* $(-\infty,1]$*. When the actual mosquito abundance in treated sectors is near the scenario of mosquito elimination, IE* $\approx$ *100%. When the observed mosquito distribution is further from the scenario of mosquito elimination versus the counterfactual distribution as estimated using the distributional synthetic control framework, IE is lower than 0. A protective treatment effect should have IE in the range of* $(0,1]$*…”*

**Major Comment 4:** Selection into Treatment Timing

The paper does not adequately address how sectors were selected for treatment and in what order. If treatment was prioritized based on mosquito abundance, dengue risk or other factors correlated with potential outcomes, this creates *confounding* that synthetic controls alone may not fully address.

Specifically, the finding that sectors with lower historical GAI exhibit higher IE (Figure 4A) could reflect: (a) A true moderating effect of baseline abundance; or (b) Selection bias if high-abundance areas were treated first and these areas have systematically different trajectories. The authors should clearly describe the treatment assignment mechanism and discuss how their identification strategy addresses potential selection on observables and unobservables (case(b)). In particular, is it possible to check if the trajectories are systematically different?

**Response: We thank the reviewer for the comment. We have initially redacted details on site selection as this has been described in a previous publication**^4^**. This was a field trial of *Wolbachia* Incompatible-insect technique coupled with sterile insect technique (IIT-SIT).** Yishun and Tampines were two large towns that were selected to adopt an expanding release strategy, where release sites were gradually expanded to adjacent neighbourhoods, to to understand behaviour, optimize release approaches in high-rise environments and the sustainability of the technology over large areas. Whereas Bukit Batok and Choa Chu Kang towns were selected on the basis of their smaller size to adopt a targeted release approach that focused releases on areas with high *Aedes aegypti* abundance and persistent dengue transmission. **Please see Bansal et al**^4^ **and the related website from the National Environment Agency, Singapore**^5^ **for the full details.**

**It is true that our outcome was *Aedes* abundance, and sites were selected based on pre-intervention data, and specifically, townships with persistently high *Aedes* abundance. This was why the distributional synthetic control method was adopted and assessed for performance here. Specifically, by balancing the entire pre-intervention distribution of outcomes between the intervention and distributional synthetic control, we controlled for baseline differences in abundance between comparator groups. Furthermore, by design, both the treated sectors and control sectors in the donor pool comprised public housing estate sectors in Singapore, which are near homogenous in nature in both spatial and time-varying characteristics, due to the extremely small spatial scale of the city-state, and are unlikely to influence the outcome measure. Our checks indicated that pre-intervention distributions of outcomes were balanced between comparator groups (See Abridged SI Figure 7 below)**

**We also plotted the distributions of mosquito abundance between the distributional synthetic controls and the actual intervention sectors in the pre-intervention period, there were no major differences in trajectories. Please see the supplementary information for this result:**

**Abridged SI Figure 7 *Ae. aegypti* abundance quantile distributions for intervened sectors in the pre-intervention period. The respective counterfactuals as estimated using distributional synthetic controls. Shaded area represents 95% confidence intervals of counterfactual quantile distribution.**

**We also visualized the relationship between post-treatment IE and historical GAI quantiles as below. There is no significant difference in IE over event-time across different historical mosquito abundance.**

**Response Figure** Intervention effectiveness (IE) trend by time-since-intervention in directly treated sectors. The colour of line represents the ranking of historical mosquito abundance in pre-intervention period among directly treated sectors. Green colour represents low mosquito abundance and red colour represents high mosquito abundance.

**Major Comment 5:** Pre-Screening of Donor Pool

The procedure of selecting the top 40 most correlated control sectors before optimization (lines 144 – 151) is not part of the original Gunsilius (2023) DSC methodology. This data-dependent pre-selection step needs to be argued better. In particular, the results might be sensitive to the choice of 40 at the cutoff. The authors should present sensitivity analyses varying the number of pre-selected controls.

**Response: We thank the reviewer for the comment. We employed a heuristic cut-off of 40 sectors due to the large number of possible donor sectors to construct the distributional synthetic control for each intervened sector, which led to computational breakdown in the DSC method.**

**To allay the reviewer’s concerns, we have now conducted a sensitivity analysis to show that increasing the number of sectors in the donor pool to 100 to 600 did not improve the weighting performance in the pre-intervention time period across sectors. Below, we appended results of this sensitivity analysis. The full results for all sectors can be found in the revised supplementary information. The text has also been amended to address this concern:**

*“Only the top 40 control sectors which were most correlated to a specific intervention sector were used for fitting the DSC for a specific intervention sector (See Supplementary materials).”*

*“(G) We conducted a sensitivity analysis on the number of control sector used in the donor pool by varying the donor pool to comprise 40, 100 or 600 control sectors. We compared the 2-Wasserstein distances of counterfactual distributions constructed by different numbers of control sectors to the treated sector’s distributions during pre-treatment period to measure the estimators’ performance. We found no significant difference in performance when varying the number of control sectors (SI Figure 10) ...”*

***Abridged SI Figure 10*** *2-Wasserstein distance between actual distribution and counterfactual distributions constructed using different donor pool sizes. Numbers on X-axis represent the last week in the 13-week interval in pre-treatment period, e.g. “−1” means “1–13 weeks before intervention”*

**Major Comment 6:** Spillover Contamination of the Donor Pool

The authors acknowledge that control sites not adjacent to treatment areas may still experience interference (lines 378-380), but dismiss this concern by citing limited dispersal of *Ae. aegypti*. However, the paper’s own results show spillover effects reaching 78% in adjacent areas. This creates a logical tension in my opinion: if *Wolbachia*-infected mosquitoes disperse sufficiently to suppress wild-type populations in adjacent sectors, they may also reach “control” sectors that are merely non-adjacent. Contamination of the donor pool would bias IE estimates downward, but the magnitude is unknown. I want the authors to address this briefly.

**Response: We thank the reviewer for raising the concern about possible spillover effects in the donor pool. In selecting controls for the donor pool, we minimized the possible spillover effect to the donor pool by selecting control sectors which were not adjacent to intervened sectors. From a meta-data analysis by Moore and Brown on the flight distance of *Ae. aegypti*, the mean average flight distance of 105.69 meters**^6^**. The average minimum distance for control sectors to the nearest treated sector in our study was 4580.74 meters, which is far larger than the average flight distance of *Ae. aegypti*. We however acknowledge that spillover contamination would bias IEs conservatively. We have now added an explanation on control site selection in the text:**

*“Similar, the donor pool consisted of sectors which did not ever receive intervention before and during the study period, and were not adjacent to release sites. To minimize the possibility of spillover contamination in control sectors comprising the donor pool, we only included control sectors which were non-adjacent to the intervention sectors for analysis. We included 676 sectors as potential control sectors in the donor pool. The average minimum distance of these control sectors to the nearest intervened sector was 4580.74 meters, which is far larger than the average flight distance (105.69 meters) of Ae. aegypti*^6^*. ”*

**Minor Comment 1:** Inference procedure

The bootstrap procedure (lines 244-247) resamples trap-level observations within sectors, which captures sampling variability and is perfectly fine. Reading the authors text, it seems like they conflate the bootstrap estimation (which exclusively deals with the randomness of the sampling), and the permutation test (which deals with the “causal interpretation”). The authors should be clearer at distinguishing these. Both are needed as they “test” different things.

**Response: We thank the reviewer for the comment. We apologize for the confusion between the two procedures. The bootstrap method is applied to construct confidence intervals for intervention effectiveness and raw reduction in GAI. The permutation test determines statistical significance of estimated intervention effects. We have now clarified the two procedures in the text and supplementary information section:**

***“****95% confidence intervals (CI) of average IE and raw reduction were calculated via bootstrap* ***f****ollowing* ***Dijcke, Gunsilius and Wright****^18^****. The bootstrap procedure was employed because as the statistics are non-linear or have unknown distributions. For each pre-treatment intervals, trap-level measurements in control sectors were resampled with replacement and new empirical quantile functions*** ${\tilde{\boldsymbol{F}}}_{\boldsymbol{j},\boldsymbol{t}}^{-\boldsymbol{1}}$ ***were estimated. New counterfactual quantile functions*** ${\tilde{\boldsymbol{F}}}_{\boldsymbol{cf},\boldsymbol{d},\boldsymbol{t}}^{-\boldsymbol{1}}$ ***were constructed based on a new weighting scheme for the intervened sector. The resampling was repeated 500 times and statistics in interest were repeatedly estimated. We obtained confidence intervals via the percentile method,*** *where the 2.5^th^ and 97.5^th^ quantile were used as the lower and upper bound of the 95% CI.”*

***“… (4) In addition to the bootstrap inference procedure, we conducted the placebo permutation test, where every control sector was reassigned to become pseudo-intervened sector to estimate its post-intervention effect, from which p-values could be obtained from the percentage of sectors that had an IE not smaller than the treated sector, to assess statistical significance.”***

***“We conducted a visual inspection of counterfactual and actual quantile functions for every treated sector during its pre-treatment period to provide a direct comparison between DSC estimates and the actual situation (SI Figure 7). The 95% confidence intervals of counterfactual quantile distributions were calculated by bootstrap procedure provided by Dijcke, Gunsilius and Wright. The resampling in bootstrap framework was repeated 500 times. We modified the calculation of resampled average weights by solving a plain sum optimization over the whole pre-treatment period rather than separately for each interval in pre-treatment period, thereby providing a joint estimation. Most of actual quantile functions fell into confidence intervals of counterfactual quantile function, indicating that DSC estimations closely fit actual situations. The result supports the reliability of counterfactual outcomes in post-treatment period.” (SI Robustness check)***

***“ Placebo permutation tests were conducted following Gunsilius. Post-intervention IEs were calculated for each control sector by removing the actual sector of interest, reassigning the control sector to be pseudo-treated sector, and repeating the analytical procedure. In each iteration, this produces an IE which is used to construct the permutation distribution. The p-value for the actual IE is taken to be the 1 minus the proportion of times the actual IE was more than the IEs in the permutation distribution... ”***

**Minor Comment 2:** Beta (3,3) sampling

The modification replacing uniform sampling with Beta(3,3) sampling (lines 158-161) is inadequately justified. Why this specific distribution? How sensitive are results to this choice?

**Response:** We thank the reviewer for raising the question regarding the sampling distribution. The specific Beta distribution parameter was chosen with reference to Li and Tolhurst’s paper^2^. They applied the Beta(3,3) distribution to alleviate the issue of extremes in quantiles with sparse observations in the weight optimization step.

We considered Beta(3,3) sampling for the weighting optimization because some empirical quantile functions had unstable upper tails in our dataset. Extreme upper-tail empirical quantiles are unstable, as the values at these quantiles rely on a small number of surveillance records. We therefore sampled based on the Beta(3,3) distribution, as it provided more stable estimates versus the uniform distribution. We have also added a sensitivity analysis on comparing the uniform vs. beta distribution, and graphical visualizations on the distance between counterfactual outcomes and the observed trend using either sampling distributions in the supplementary information section for all treated sectors (abridged figure provided below). We found that differences are small and negligible in using either sampling scheme. Please see the revised supplementary information for these results:

*“(B) We conducted a sensitivity analysis check on the choice of sampling distributions in the weight optimization step. We considered the uniform distribution and Beta(3,3) distribution to randomly select quantiles. We graphically visualized the difference in outcomes in the post-intervention period using either sampling distribution to examine the influence of the sampling distribution on our results…” (SI Sensitivity Check (B))*

***Abridged SI Figure 21*** *Difference in values between counterfactual quantile functions computed via sampling using the beta or uniform distribution in studying Ae. aegypti abundance. Red line represents the raw GAI difference between actual and counterfactual quantile distributions calculated from Beta(3,3) sampling distribution. The blue line represents the raw GAI difference between actual and counterfactual quantile functions calculated from uniform sampling distribution. Each line represents one pre-treatment time interval in the pre-treatment period.*

**Minor Comment 3:** Negative spillover IE

The reported spillover IE of−1.29% in weeks 1–52 (line 292) deserves explanation. Does this suggest the intervention temporarily increased mosquito abundance in adjacent areas, perhaps through displacement? Or is this an artifact of estimation error? The biological interpretation matters.

**Response:** We thank the reviewer for the comment. The formula $IE_{d,t}= \frac{\hat{W_{2}}\left( F_{cf,d,t}^{-1}, F_{perfect}^{-1} \right)- \hat{W_{2}}(F_{d,t}^{-1}, F_{perfect}^{-1})}{\hat{W_{2}}(F_{cf,d,t}^{-1}, F_{perfect}^{-1})}$ will yield a negative quantity if the observed mosquito distribution is higher than the counterfactual outcome distribution and not due to an estimation error. This small drop is likely due to sampling noise in the early intervention period, as alluded to by the large confidence intervals surrounding this estimate (IE: −1.29%, 95% CI: −14.67% – 10.24%)~~.~~

**Minor Comment 4:** Core vs. buffer distinction

The distinction between core sectors (no border with controls) and buffer sectors (border with controls) is introduced in lines 203-205 but not systematically exploited in the results. Given the paper’s focus on spatial heterogeneity, separate analyses for these categories would be informative.

**Response:** We thank the reviewer on the suggestion of separately analysing the difference between two types of treated sectors. We added a detailed interpretation on the differences of IE trend between treated sectors in core and buffer area:

*“We also computed the average IE between core sectors and buffer sectors. We did not find significant IE difference across the two year post-intervention period (See supplementary materials).”*

*“We further studied possible treatment effect heterogeneity by comparing IE over the intervention time between buffer and core sectors. However, we found similar trajectories in treatment effect. During the first 52-week treatment, average IE in core areas increased from 28.09% (95%CI: 17.78% – 40.81%) to 62.63% (95%CI: 48.41% – 81.78%), while average IE in buffer areas increased from 20.71% (95%CI: 11.52% – 29.65%). However, these effects were not different statistically (Welch’s T-test p-val >0.05). ”*

***SI Figure 2***

(A) *IIT-SIT intervention effectiveness to reduce Ae. aegypti abundance by time-since-intervention in core versus buffer sectors*
(B) *IIT-SIT intervention effectiveness to reduce Ae. aegypti abundance by calendar time in core versus buffer sectors*

**Minor Comment 5:** Writing quality

The manuscript contains numerous grammatical errors and typos that impede readability. Examples include:

• “Heterogenous” should be “Heterogeneous” throughout.

• Line 54: “complements environmental management (REF)” – missing reference.

• Line 189: “deviatesfrom” should be “deviates from.”

• Various instances of inconsistent capitalization and punctuation.

**Response:** We thank the reviewer for pointing out the grammatical errors. We have now checked throughout the manuscript for these errors and have amended as follows:

1. We have changed “Heterogenous” to “Heterogeneous” throughout the text.
2. We have removed the “(REF)” in L54
3. Line 189: The original text is “*deviates from*”. We have now added the spacing in L189
4. We have now extensively revised the text on punctuation and capitalization

**Reviewer #2**

**Comment 1:** I have some concern on the novelty of this new work. It looks like most key findings have been published in their previous work Ref [4]. I am not sure if the current study gives more insights on the IIT-SIT interventions.

**Response: We thank the reviewer for highlighting the need to clarify the novelty of our work. Briefly, our previous work demonstrated the effectiveness of IIT-SIT in suppressing adult female *Ae. aegypti* populations and highlighted the temporal trend of intervention effectiveness (IE).**

Here, IE estimates yielded by DSC indicated treatment effect heterogeneity on both spatial and temporal scales. In contrast, the canonical SCM yields pointwise estimated IE and the temporal heterogeneity of treatment effects. In the canonical SCM, the intervention effectiveness of *Wolbachia* is evaluated via average mosquito abundance in sectors over time, however, this disregards the finer-scale trap-level information in sectors. Whereas DSC yields intervention effectiveness estimates using the entire quantile of observed mosquito abundance within sectors which contains more information about variation and spread of data in those evaluated sectors. By obtaining counterfactual quantile functions as the comparator group to the observed quantile function in intervention sites, IE estimates can be expressed in other statistics, such as the IE over different mosquito abundance quantiles, and help tease out heterogeneous treatment effects over smaller-scale spatial scales. Leveraging this method, we could also estimate whether elimination of ***Ae. aegypti* was possible. We added now added detailed explanations in the methods and results section to outline the novelty of our work:**

*“IE estimates yielded by DSC indicated treatment effect heterogeneity on both spatial and temporal scales. In contrast, the canonical SCM yields pointwise estimated IE and the temporal heterogeneity of treatment effects. In the canonical SCM, the intervention effectiveness of Wolbachia is evaluated via average mosquito abundance in sectors over time, however, this disregards the finer-scale trap-level information in sectors. Whereas DSC yields intervention effectiveness estimates using the entire quantile of observed mosquito abundance within sectors which contains more information about variation and spread of data in those evaluated sectors. By obtaining counterfactual quantile functions as the comparator group to the observed quantile function in intervention sites, IE estimates can be expressed in other statistics, such as the IE over different mosquito abundance quantiles, and help tease out heterogeneous treatment effects over smaller-scale spatial scales.”*

***“By comparing the IEs over different quantiles, we found that in directly treated sectors, quantile IEs around the 25^th^ quantile were higher than that at other quantiles. Quantile IEs around the 0^th^ quantile were lower and gradually increased as quantiles increased. Comparing quantile IE between the 25^th^ – 49.9^th^ quantile and 75^th^ – 100^th^ quantile , the maximum quantile IE difference decreased from 61.33% in 1 – 13 weeks after intervention to 29.62% in week 105 – 117, and the minimum quantile IE difference decreased from 24.40% to −0.7%. This showed that IE reduced quickly at higher GAI quantiles at the beginning of treatment, and treatment effect was lower in moderate mosquito abundance areas in the sector. Longer treatment times could however shrink the difference on treatment effectiveness between mosquito quantiles…”***

**(Part for Figure 4) *“*** $IE_{d,t}$ *was significantly higher in sectors with lower historical pre-treatment GAI . However, as the intervention time increased, IEs over different historical mosquito quantiles converged to a similar level (Figure 4A). Intervention effectiveness increased with more intervention time with little heterogeneity across intervened townships (Figure 4B). Areas with historical high mosquito populations required 52 weeks to reach 75% suppression while areas with low historical mosquito populations required 26 weeks.****”***

**Comment 2:** The presentation is difficult to follow. The authors are good at summarizing technical details. However, this makes readers feel distraction as it’s not easy to get their points. To be honest, the format of this paper is more like a technical summary instead of research article. I would suggest the authors carefully edit the manuscript according to the format and guidelines of PLoS Computational Biology.

**Response: We thank the reviewer for the comment. We have now extensively revised the manuscript to include more results stemming from the technical details to provide more context to our results. Please see point-by-point responses to the specific concerns highlighted below.**

**Comment 3:** It is unclear to me why the DSC approach is appropriate and the best approach to evaluate the causal relationship in the study setting. Given the presence of other confounding factors such as climate factors, intervention measures induced by COVID control policies, human population awareness of infectious pathogens, I’m nervous about the claim on the inference of causal relationship.

**Response: We thank the reviewer for pointing out the importance of controlling for confounders during the study period. In general, the canonical synthetic control method is robust to observed and unobserved time-varying confounders if sufficiently long pre-intervention data is provided to obtain the counterfactual**^8,9^**, and a close fit between treated and synthetic control outcomes over a long pre-intervention period implies balance in both observed and unobserved confounders relevant to the outcome, as these are confounders which have likely produced the trends in outcome. We have shown previously in SI that the fit provided under the DSC framework was good (abridged below):**

**Abridged SI Figure 7 *Ae. aegypti* abundance quantile distributions for intervened sectors in pre-intervention period and their respective counterfactuals as estimated using distributional synthetic controls. Shaded area represents 95% confidence intervals of counterfactual quantile distribution.**

**Furthermore, our donor pool included a large number of control sectors (n=662) to construct the counterfactual outcomes for the 91 treated sectors in the post-intervention period. As observed or unobserved confounders drive the outcomes in the pre-intervention period, by balancing the trends in the outcome over the pre-intervention period, it is likely that our framework balances observed and unobserved counfounders as well in the same period**^9^**. Furthermore, by design, both the treated sectors and control sectors comprising the donor pool comprised public housing estate sectors in Singapore, which are near homogenous in nature in both spatial and time-varying characteristics, due to the extremely small spatial scale of the city-state, and are unlikely to influence the outcome measure.**

**Our intervention effectiveness estimates also comprised estimates across calendar time. This meant that both the counterfactual and observed outcomes were contrasted against in the same calendar time. Consequentially, nation-wide transitory shocks, such as the COVID-19 pandemic period, are already accounted for in computation of intervention effectiveness estimates, with no major differences estimated under this aggregation.**

**Comment 4:** Line 158, the authors mentioned “We used the procedure of Li and Tolhurst by replacing uniform random sampling of quantile functions to beta distribution random sampling [16].” Given that the choice of sampling distribution can impact the estimated synthetic counterfactual, the authors are recommended to perform a sensitivity analysis to see if the findings remain robust if a standard uniform distribution or different Beta parameters are used.

**Response: We thank the reviewer for the comment.**

We considered Beta(3,3) sampling for the weighting optimization because some empirical quantile functions had unstable upper tails in our dataset. Extreme upper-tail empirical quantiles are unstable, as the values at these quantiles rely on a small number of surveillance records. We therefore sampled based on the Beta(3,3) distribution, as it provided more stable estimates versus the uniform distribution. We have also added a sensitivity analysis on comparing the uniform vs. beta distribution, and graphical visualizations on the distance between counterfactual outcomes and the observed trend using either sampling distributions in the supplementary information section for all treated sectors (abridged figure provided below). We found that differences are small and neglectable in using either sampling scheme. Please see the revised supplementary information for these results:

*“(B) We conducted a sensitivity analysis check on the choice of sampling distributions in the weight optimization step. We considered the uniform distribution and Beta(3,3) distribution to randomly select quantiles. We graphically visualized the difference in outcomes in the post-intervention period using either sampling distribution to examine the influence of the sampling distribution on our results” (SI Sensitivity Check (B))*

Changing sample distribution from uniform distribution to Beta(3,3) distribution did not drastically affect optimization results:

***Abridged SI Figure 21*** *Difference in values between counterfactual quantile functions computed via sampling using the beta or uniform distribution in studying Ae. aegypti abundance. Red line represents the raw GAI difference between actual and counterfactual quantile distributions calculated from Beta(3,3) sampling distribution. The blue line represents the raw GAI difference between actual and counterfactual quantile functions calculated from uniform sampling distribution. Each line represents one pre-treatment time interval in the pre-treatment period.*

**Comment 5:** Line 150, "Only the top 40 control sectors which were most correlated to a specific intervention sector were used for fitting the DSC”. It is unclear whether this "top 40" threshold might introduce a selection bias that favors control sectors with similar local environmental shocks rather than those representing the broader counterfactual trend.

**Response: We thank the reviewer for raising the concern regarding control sector selection. We thank the reviewer for the comment. We employed a heuristic cut-off of 40 sectors due to the large number of possible donor sectors to construct the distributional synthetic control for each intervened sector, which led to computational breakdown in the DSC method.**

**To allay the reviewer’s concerns, we have now conducted a sensitivity analysis to show that increasing the number of sectors in the donor pool to 100 to 600 did not improve the weighting performance in the pre-intervention time period across sectors. Below, we appended results of this sensitivity analysis. The full results for all sectors can be found in the revised supplementary information. The text has also been amended to address this concern:**

*“(G) We conducted a sensitivity analysis on the number of control sector used in the donor pool by varying the donor pool to comprise 40, 100 or 600 control sectors. We compared the 2-Wasserstein distances of counterfactual distributions constructed by different numbers of control sectors to the treated sector’s distributions during pre-treatment period to measure the estimators’ performance. We found no significant difference in performance when varying the number of control sectors (SI Figure 10) ...”*

***Abridged SI Figure 10*** *2-Wasserstein distance between the actual and counterfactual distributions constructed using different donor pool sizes. Numbers on X-axis represent the last week in the 13-week interval in pre-treatment period, e.g. “−1” means “1 – 13 weeks before intervention”*

**Reviewer #3 comments**

**Major Comment 1:** Species-specific elimination definition

No elimination = no *Aedes* *aegypti*/*albopictus*? Why is *Aedes albopictus* considered here, while the released mosquitoes are *Aedes* *aegypti*, and the objective is to measure the impact on Aedes *aegypti*? Would removing the *Aedes albopictus* count increase the impact estimate? Surely the probability number shows a very low probability of elimination, but shouldn't the calculation consider Aedes *aegypti* only to make the elimination probability calculation exclusive for *Aedes aegypti* (since the trial released *Aedes aegypti* only)?

**Response: We thank the reviewer for the comment. *Ae. aegypti* is the primary vector of dengue virus. The study focuses on the impact of *Wolbachia* interventions on *Ae. Aegypti* abundance. We have now removed the portions on the elimination probability for *Aedes* *albopictus*. However, in our prior study, as we have found that *Wolbachia* interventions had effects on *Ae. aegypti* and *albopictus* abundance, we have retained the results for the other analyses concerning either outcome. We apologize for the lack of clarity and have amended the text accordingly:**

***“****We further analysed if the IIT-SIT intervention led to an increase in the percentages of traps recording no wild-type female Ae. aegypti mosquitoes. This was done to ascertain if elimination of wild-type female Ae. aegypti mosquitoes was possible given sufficient intervention time. We found that the mean increase in the percentages of traps recording no wild-type female Ae. aegypti mosquitoes was around 6.77% (95%CI: 5.49% – 8.12%) – 33.01%(95%CI: 29.50% – 36.57%) in the 1 to 124 weeks after interventions began (Figure 5A).”*

**Major Comment 2:** Trial protocol

What is not clear here is how many times the mosquitoes were released during the observation period? What is the density of the release? Are they all the same for each sector? Any frequency and periodicity that may affect the observations and thus the analysis? Was it done one time at the beginning? And nothing was done regarding the release during the observation period? I understand that previous studies have discussed this, but it would help readers unfamiliar with this trial by providing guidance on the trial's designs, thereby helping them connect it more easily to the results.

**Response: We thank the reviewer for the comment. We apologise for the confusion; this section has been initially redacted as they have been reported in our prior study. We have now explained the trial release protocols in the methods section. The density of the release is uniform across sectors:**

***“****Entomological surveillance data was collected from Gravitraps which were designed to attract and catch gravid female Aedes mosquitoes. Each trap was measured twice a week, and can be individually geo-located to the postal code, representing a single apartment block. Surveillance data from Epidemiological Week (EW) 1 2019 to EW26 2022 was considered to evaluate the heterogeneous impact of Wolbachia IIT-SIT on wild-type Aedes aegypti and albopictus populations. Data after EW26 2022, was excluded due to rollout of large-scale randomised controlled trials in the study setting in most high-risk locations in Singapore, which made construction of the synthetic control donor pool challenging^12,13^. The spatial resolution used for analyses were traps within sectors, which comprise 10 – 20 public housing apartment blocks each and measure an average of approximately 0.088 km^2^. Sectors are used for administration of surveillance and control for environmental infectious diseases in Singapore. To ensure that sufficient data was available to construct distributional synthetic controls, only sectors which had a pre-treatment period of 52 weeks or more were included, with 91 sectors included in the study as treated sectors or intervened sectors. Similar, the donor pool consisted of sectors which did not ever receive intervention before and during the study period, and were not adjacent to release sectors. To minimize the possibility of spillover contamination in control sectors comprising the donor pool, we only included control sectors which were non-adjacent to the intervention sectors for analysis. We included 676 sectors as potential control sectors in the donor pool. The average minimum distance of these control sectors to the nearest intervened sector was 4580.74 meters, which is far larger than the average flight distance (105.69 meters) of Ae. aegypti^14^.*

***Releases of Wolbachia-infected male Ae. aegypti mosquitoes were conducted at fixed designated locations in the high-rise apartment blocks. There are 6 to 12 release locations which equally distributed among ground level, middle floors (levels 5 – 6) and high floors (levels 10 – 11) of each apartment blocks. To maintain the density of intervention and reduce effect variation, Wolbachia-infected male mosquitoes were released twice a week in intervened sites. The density of release is uniform across sectors. Treatment for every sector started at different weeks and ended at the same week (EW26, 2022).”***

**Major Comment 3:** Data interpolation methods for odd weeks

Data were collected every two weeks, but how do you generate data for odd weeks (i.e., observation weeks start from week 1 and end in week 13)? How does the algorithm calculate the data to account for week 13, as, from my understanding, observations must be made at week 14?

**Response: We thank the reviewer for pointing out the error. Trap data were collected twice a week rather than every two weeks. No modifications were made on the original data. The text is revised as follows:**

***“****Entomological surveillance data was collected from Gravitraps which were designed to attract and catch gravid female Aedes mosquitoes. Each trap was measured twice a week, and can be individually geo-located to the postal code, representing a single apartment block.” (Line 92-94)*

**Major Comment 4:** Synthetic control site reuse and township boundaries

Can a control from the donor pool be used multiple times for different intervention sites if they have high correlations for those sites relative to others? Or can each control only be used for a single intervention site? If they can be used multiple times, are there instances in which a single site from the donor pool served as a synthetic control across multiple intervention sites, thereby driving the results? Or was the weighting different for this site across those intervention sites? Can a site in a different township serve as a control site? If yes, will this affect the results?

**Response:** We thank the reviewer for the comment. A control sector from the donor pool may be selected multiple times for different intervened sectors, and has to be in a different township with geographical borders being non-adjacent to intervention sites. There is no restriction on the number of times a control sector can contribute to synthetic controls for different intervention sectors. In general, the controls selected to contribute to distributional synthetic controls and the distributional synthetic weights for each control sectors are highly varied. We found that there are no instances of specific control sectors being highly correlated to multiple intervention sectors. Please see the supplementary information section for a full list of weights for each intervention sector (SI Table 1 – 7).

**Abridged SI Table 1** Donor weights for the 9 Bukit Batok sectors (total 9). Only sectors with any contribution to construct counterfactual distribution for any intervened sector are listed.

**Major Comment 5:** Pseudo-intervention definition

What does assigning a pseudo-intervention mean? Does that mean just assigning them as intervention sites (without actually changing any values at those sites), and hence the hypothesis is that there will be no difference? Or some intervention efficacy assigned to these sites (with known values) to test the hypothesis?

**Response:** We thank the reviewer for raising the question regarding pseudo-intervention.

In placebo tests:

(**1**) a pseudo-intervention time interval pertains to designating a period prior to the actual treatment start time to be considered as an intervention period. This pseudo-intervention time was used in the in-time placebo test to demonstrate that our analytical strategy did not detect intervention effects in the period where no intervention was in place, and that no pre-trends were influencing intervention effectiveness estimates in the post-intervention period in the treated sites.

(**2**) a pseudo-intervention sector refers to a control sector in donor pool that is considered an intervened sector when we are conducting in-space placebo tests. This test was used to demonstrate that our analytical strategy did not detect intervention effects in the control sites which may be influencing intervention effectiveness estimates in the treated sites.

We have now added the following text below for clarity:

*“(A) In-time placebo checks were done, with pseudo-treatment start dates for actual intervention sectors done 26 – 52 weeks prior to actual treatment. Estimated intervention effectiveness on Ae. aegypti population was far smaller, with estimated median IEs of 1.14% and −0.53% for the pseudo-intervention period starting from 26 and 52 weeks prior to the start of the actual intervention respectively (SI Figure 5A). Raw reductions in Ae. aegypti population in direct-intervened sectors between placebo quantile distribution and actual quantile distribution ranged from −0.442 – 0.162 and −0.377 – 0.189 for 26 and 52 weeks prior to the intervention, which were far smaller than the actual intervention effect.*

*(B) In-space placebo tests were conducted by removing the actual treated sectors, and repeating our analytical procedure by reassigning control sectors to be the pseudo-treated sector. We found that estimated raw reduction on each quantile of Ae. aegypti population was far smaller, and ranged from −0.008 – 0.017 for the pseudo-intervention control sectors (SI Figure 6A).*

*(C) We repeated the in-time and in-space placebo checks for the endpoint of Ae. albopictus abundance and found that our analytical strategy did not reproduce significant intervention effectiveness estimates for the endpoint of Ae. albopictus abundance in pseudo-intervention sectors or time periods (SI Figure 5C, 6C).*

*(D) We repeated the in-time placebo checks for the spillover impact of Ae. aegypti and Ae. albopictus abundance and found that our analytical strategy did not reproduce significant intervention effectiveness estimates for the endpoint of Ae. albopictus abundance in pseudo-intervention time periods in spillover locations (SI Figure 5D, 5F, 6D, 6F).”*

**Major Comment 6:** Table 1 discrepancy

In Table 1, why are the numbers for Direct not equal to the sum of Core and Buffer? Isn't the Direct one supposed to mean accounting for both based on the definition?

**Response:** We thank the reviewer for the comment. Core sectors have borders which do not touch non-intervention sectors, whereas buffer sectors are sectors which have shared borders with intervention sectors. **A treated sector may be converted from a “buffer” sector to a “core” sector over time. This is most apparent in the case of Tampines and Yishun,** which were two large towns that adopted an expanding release strategy, where sectors adjacent to releases gradually adopt *Wolbachia* intervention (See figure below for visualization)**. Traps may therefore be counted as both “Buffer” and “Core” sectors in a certain time period. We have now amended the text for clarity:**

|  | *EW8–EW26 2020* | *EW27–EW53 2020* | *EW1–EW26 2021* | *EW27–EW52 2021* | *EW1–EW26 2022* | *Total* |
| --- | --- | --- | --- | --- | --- | --- |
| *Sites* | *Traps (Sectors)* | | | | | |
| *Core^1^* | *648 (7)* | *1144 (13)* | *2016 (23)* | *2555 (33)* | *5358 (69)* | *5619 (69)* |
| *Buffer^2^* | *2526 (26)* | *3148 (37)* | *2843 (37)* | *2798 (36)* | *2795 (34)* | *4809 (50)* |
| *Direct^3^* | *2987(31)* | *3902 (46)* | *4402 (55)* | *5066 (64)* | *7287 (91)* | *8239 (91)* |
| *Spillover^4^* | *2233 (23)* | *2118 (28)* | *1988 (25)* | *1672 (21)* | *1531 (20)* | *3539 (40)* |
| *Control^5^* | *45784 (662)* | *45799 (662)* | *46688 (662)* | *46280 (662)* | *46253 (662)* | *51457 (662)* |

***Table 1*** *Number of traps and study sectors (in brackets) used for analysis.*

*^1^Directly-treated sectors which did not share borders with control sectors.*

*^2^Directly-treated sectors which shared borders with control sectors.*

*^3^Directly treated sites.*

*^4^Spillover sites refer to locations which shared borders with treated sectors but were not directly treated.*

*^5^Control sites which do not share borders with directly treated sectors and are not treated.*

**Note that traps in a sectors may be counted in both “Core” and “Buffer” sites when the treated sectors changed from being designated a buffer to core sector during that specific time interval*

***SI Figure 11*** *Intervention effectiveness of IIT-SIT on Ae. aegypti abundance by sector. Columns represent the respective time periods of EW27 – 53 2020, EW1 – 26 2021, EW27 – 52 2021 and EW1 – 26 2022. Rows represent each township. From top to bottom: Bukit Batok, Choa Chu Kang, Tampines and Yishun townships are represented*

**Major Comment 7:** Seasonal mosquito abundance heterogeneity and adjustment

Is there any seasonal heterogeneity in mosquito abundance across the study sites? If yes, how did the IE calculation account for this? In the method, it was noted that calendar time and event time adjustments were performed. But it is not clear whether there's seasonality in mosquito abundance, whether the adjustment accounts for it, and whether the IE is, importantly, not biased by seasonality at the sites that adopted treatment later in the study.

**Response:** We thank the reviewer for the concern regarding seasonal heterogeneity. **Both the treated sectors and control sites in the donor pool comprised public housing estate sectors in Singapore, which are near homogenous in nature in both spatial and time-varying characteristics, due to the extremely small spatial scale of the city-state, and are unlikely to influence the outcome measure. It is also a tropical, equatorial city-state** with relatively stable temperature and rainfall. Seasonal variation is therefore expected to have minimal impact on the treatment effect.

We have however applied IE aggregations by both time-since-intervention and calendar time, so that potential differences in treatment effect can be summarized over different time periods. IE increased gradually over time-since-intervention and calendar time, implying that seasonal variations do not influence estimates significantly:

*“By comparing the quantile functions between intervention sectors and the respective distributional synthetic controls, we found that the direct intervention effectiveness (IE) was greater in locations that were subject to longer intervention periods (Figure 2A). From 1 – 52 weeks post intervention, the average 13-week IE ranged from 24.04% (95%CI: 16.61% – 30.85%) to 71.51% (95%CI: 65.02% – 77.18%). In contrast, in 53 – 124 weeks post intervention, the average 13-week IE ranged from 80.04% (95%CI: 76.26% – 83.46%) to 86.08% (95%CI: 82.89% – 88.78%). Raw reductions in abundance were also greater in locations that were subject to longer intervention periods (Figure 2C).”*

***“By comparing the IEs over different quantiles, we found that in directly treated sectors, quantile IEs around the 25^th^ quantile were higher than that at other quantiles. Quantile IEs around the 0^th^ quantile were lower and gradually increased as quantiles increased. Comparing quantile IE between the 25^th^ – 49.9^th^ quantile and 75^th^ – 100^th^ quantile , the maximum quantile IE difference decreased from 61.33% in 1 – 13 weeks after intervention to 29.62% in week 105 – 117, and the minimum quantile IE difference decreased from 24.40% to −0.7%. This showed that IE reduced quickly at higher GAI quantiles at the beginning of treatment, and treatment effect was lower in moderate mosquito abundance areas in the sector. Longer treatment times could however shrink the difference on treatment effectiveness between mosquito quantiles…”***

*“IE in later study periods (EW1 – 26, 2022) was also higher than that in earlier study periods (EW8 – 53, 2020). Mean relative reductions in Ae. aegypti abundance were heterogeneous in the initial study period (i.e. earlier e-weeks) and earlier timepoints that a trap location is intervened, but gradually converged at around 1-year post intervention with more than 80% suppressive effectiveness on Ae. aegypti abundance (See supplementary materials).”*

**Major Comment 8:** Core versus Buffer differentiation

I feel more elaboration is needed in the Core and Buffer areas' IE. From my understanding, it is shown in Figure 3. However, there's little elaboration on whether there's any difference in IE between those places, even though the study was designed to detect it. In general, figure results were not elaborated clearly and completely and some of them were left to readers’ interpretation. I suggest the authors to provide more elaborations on the figures shown in the manuscript to guide interpretations.

**Response:** We thank the reviewer for the comment. We have now elaborated on core vs. buffer area sector IEs:

*“We further studied possible treatment effect heterogeneity by comparing IE over the intervention time between buffer and core sectors. However, we found similar trajectories in treatment effect. During the first 52-week treatment, average IE in core areas increased from 28.09% (95%CI: 17.78% – 40.81%) to 62.63% (95%CI: 48.41% – 81.78%), while average IE in buffer areas increased from 20.71% (95%CI: 11.52% – 29.65%). However, these effects were not different statistically (Welch’s T-test p-val >0.05).”*

*“We also computed the average IE between core sectors and buffer sectors. We did not find significant IE difference across the two year post-intervention period (See supplementary materials).”*

***SI Figure 2***

(A) *IIT-SIT intervention effectiveness to reduce Ae. aegypti abundance by time-since-intervention in core versus buffer sectors*
(B) *IIT-SIT intervention effectiveness to reduce Ae. aegypti abundance by calendar time in core versus buffer sectors*

**Major Comment 9:** Comparison with prior studies

I think in the discussion, it may help to provide context for the estimates of IE from previous studies using similar data (with a different approach). Are there any differences in estimates when understanding the distributional IE rather than just the average, and when accounting for smaller-scale spatial-level data? Are the overall estimates ultimately the same (i.e., township-level estimates)?

**Response:** We thank the reviewer for raising the question on the differences of results between two methods. We have added discussion about comparison of IE between SCM and DSC and benefits of using IE estimates calculated by DSC.

*“IE estimates yielded by DSC indicated treatment effect heterogeneity on both spatial and temporal scales. In contrast, the canonical SCM yields pointwise estimated IE and the temporal heterogeneity of treatment effects. In the canonical SCM, the intervention effectiveness of Wolbachia is evaluated via average mosquito abundance in sectors over time, however, this disregards the finer-scale trap-level information in sectors. Whereas DSC yields intervention effectiveness estimates using the entire quantile of observed mosquito abundance within sectors which contains more information about variation and spread of data in those evaluated sectors. By obtaining counterfactual quantile functions as the comparator group to the observed quantile function in intervention sites, IE estimates can be expressed in other statistics, such as the IE over different mosquito abundance quantiles, and help tease out heterogeneous treatment effects over smaller-scale spatial scales.”*

We have also compared our output with that of prior study results on township-level IE estimation, we observed a similar IE trend but lower IE values across all four intervened townships. However, the IE values from two studies cannot be directly compared as the canonical synthetic control method used mean abundance over time to evaluate treatment effects, but the DSC method used in our text used the entire distribution of recorded mosquito abundance to obtain IEs. In our study, the IE estimates were lower compared to the original study. Please see the tabulated summary of results below:

| Time period | 1 – 6 months | 7 – 12 months | 13 – 18 months | 19 – 24 months | > 24 months |
| --- | --- | --- | --- | --- | --- |
| IE | **Bukit Batok** | | | | |
| Prior study (SCM) | 49.55% | 92.34% | 95.25% | 95.47% | 94.77% |
| DSC | 22.70% | 85.84% | 88.86% | 89.32% | 90.00% |
|  | **Choa Chu Kang** | | | | |
| Prior study (SCM) | 60.11% | 82.60% | 90.09% | 94.60% | 96.13% |
| DSC | 41.52% | 70.07% | 79.06% | 88.67% | 93.10% |
|  | **Tampines** | | | | |
| Prior study (SCM) | 52.91% | 68.32% | 80.98% | 82.92% | 90.07% |
| DSC | 21.73% | 39.87% | 67.39% | 69.76% | 81.87% |
|  | **Yishun** | | | | |
| Prior study (SCM) | 59.72% | 87.84% | 92.44% | 91.58% | 93.29% |
| DSC | 36.61% | 65.35% | 83.65% | 84.69% | 79.84% |

**Response Table** Result of Intervention Efficacy (IE) of 4 treated townships during the post-intervention periods using the SCM and DSC methodologies.

**Major Comment 10:** Future methodological and trial design guidance

I think the discussions could also be improved by relating the results to other study contexts and by outlining what to develop further. Since the method is also novel, can the authors suggest how data collection or study design could be improved to better apply the DSC method in the future (e.g., so less distributional adjustment is needed, and trial designs will then support the establishment of this type of analysis)? Hence, can the method then be applied with fewer assumptions and fewer smoothing steps?

**Response:** We thank the reviewer for raising concern regarding trial design improvement. We have added these recommendations into the limitations and strengths paragraph of the discussion section:

*“Our study has several strengths. Our analysis relied on a large, nationwide, routinely collected surveillance database on mosquito abundance. By using trap-level records, we could obtain and construct DSCs which enabled us to understand the heterogeneous effects of Wolbachia-Aedes on wildtype Aedes abundance, and understand if elimination of wildtype mosquitoes was possible. This analysis was supported by extensive internal validity checks – including in-space, in-time placebo checks, and additional inferential procedures such as permutation testing. These procedures enabled us to allay concerns that estimates of intervention effectiveness were spurious or due to poor model predictive performance. There are however certain limitations. Despite the extremely high resolution of our data, we tagged trap-level measurements into 13-week intervals at the sector level, which enabled us to understand heterogeneous intervention effectiveness across sites, but does not allow us to exactly identify the exact traps locations where wild-type mosquito populations do not have high IEs or elimination. Due to the short study period and initiation of a large-scale cluster randomised controlled trial in 2022^12,13^, further evaluation of entomological effectiveness in historically high-abundance locations which have lower intervention effectiveness cannot be done.* ***In future studies, increasing the number of samples via more intensive surveillance can also enable analyses at a finer temporal resolution and potentially help DSC estimate finer-scale treatment effects.*** *Lastly, construction of DSCs assumed that non-intervened locations which were not adjacent to intervention sectors did not experience partial interference from intervention sectors. Yet,* ***DSC relies on the stable unit treatment value assumption (SUTVA), requiring that the donor pool not be affected by treatment effects. This assumption is violated when the intervention generates spillover effects in the donor pool, which may lead intervention effectiveness estimates being conservative.*** *While previous studies have shown limited dispersal for the Ae. aegypti vector in Singapore^19^, interference of control sites may bias estimated intervention effectiveness downwards.”*

**Minor point:**

Abstract, Line 38: GAI hasn't been defined;

Line 54: Not sure if (REF) there refers to a planned reference to be added or an acronym for previous words;

Line 356: "Conventional" came up twice;

Terminology: For consistency, write mosquito species as Ae. aegypti throughout rather than just "aegypti";

Figure 2: Suggest that the scale for the y-axis in panels C and D should be made similar for ease of comparison

**Response:** We thank the reviewer for pointing out the text errors. These have now been amended:

1. Abstract, Line 38: *“However, IE converged across sites with different historical mosquito abundance as intervention time increased.”*
2. Line 54: *“IIT-SIT offers several advantages over conventional vector control strategies such as insecticide-based approaches, and complements environmental management.”* (REF) is removed.
3. Line 356: *“This also highlights the potential need to integrate Wolbachia-based suppression with other conventional control measures, such as…”* One of the two ‘conventional’ is removed.
4. Terminology: All mosquito species are changed from “*aegypti*” to “*Ae. aegypti*”
5. Figure 2: Revised based on reviewer’s suggestion.

**Reference**

1.	Dijcke, D. V., Gunsilius, F. & He, S. DiSCos: Distributional Synthetic Controls Estimation. (2026).

2.	Li, Y. & Tolhurst, T. N. Adoption of Genetically Engineered Seeds in China: Predicting Treatment Effects on the Crop-Yield Distribution by Synthetic Control. *2024 Annual Meeting, July 28-30, New Orleans, LA* https://ideas.repec.org//p/ags/aaea22/343921.html (2024).

3.	Dijcke, D. V., Gunsilius, F. & Wright, A. Return to Office and the Tenure Distribution. Preprint at https://doi.org/10.48550/arXiv.2405.04352 (2024).

4.	Bansal, S. *et al.* Effectiveness of *Wolbachia*-mediated sterility coupled with sterile insect technique to suppress adult *Aedes aegypti* populations in Singapore: a synthetic control study. *The Lancet Planetary Health* **8**, e617–e628 (2024).

5.	*Wolbachia*-Aedes Mosquito Suppression Strategy. https://www.nea.gov.sg/corporate-functions/resources/research/environmental_health_institute/wolbachia-aedes-mosquito-suppression-strategy.

6.	Moore, T. C. & Brown, H. E. Estimating *Aedes aegypti* (Diptera: Culicidae) Flight Distance: Meta-Data Analysis. *J Med Entomol* **59**, 1164–1170 (2022).

7.	Gunsilius, F. F. Distributional Synthetic Controls. *Econometrica* **91**, 1105–1117 (2023).

8.	Abadie, A., Diamond, A. & Hainmueller, J. Synthetic Control Methods for Comparative Case Studies: Estimating the Effect of California’s Tobacco Control Program. *Journal of the American Statistical Association* **105**, 493–505 (2010).

9.	Abadie, A., Diamond, A. & Hainmueller, J. Comparative Politics and the Synthetic Control Method. *American Journal of Political Science* **59**, 495–510 (2015).
