# Supplementary material for: Heterogeneous suppressive effect of Wolbachia incompatible insect technique coupled with sterile insect technique across time and historical Ae. aegypti abundance - using distributional synthetic controls
Source: PLoS Comput Biol. 2026 Jun 22;22(6):e1014355. doi: 10.1371/journal.pcbi.1014355 (PMC13336471; doi:10.1371/journal.pcbi.1014355)
Supplement: Attachment — Submitted filename: Full_response_Apr08.docx [file pcbi.1014355.s006.docx]

**Editorial Letter**

**Comment**: Dear Dr. Zhai,

Thank you for submitting your manuscript to PLOS Computational Biology. After careful consideration, we feel that it has merit but does not fully meet PLOS Computational Biology's publication criteria as it currently stands. Therefore, we invite you to submit a revised version of the manuscript that addresses the points raised during the review process. In particular, there are a couple of minor comments that need to be addressed before the paper can be accepted.

Please submit your revised manuscript by May 31 2026 11:59PM. If you will need more time than this to complete your revisions, please reply to this message or contact the journal office at ploscompbiol@plos.org. When you're ready to submit your revision, log on to https://www.editorialmanager.com/pcompbiol/ and select the 'Submissions Needing Revision' folder to locate your manuscript file.

Please include the following items when submitting your revised manuscript:

* A letter that responds to each point raised by the editor and reviewer(s). You should upload this letter as a separate file labeled 'Response to Reviewers'. This file does not need to include responses to formatting updates and technical items listed in the 'Journal Requirements' section below.

* A marked-up copy of your manuscript that highlights changes made to the original version. You should upload this as a separate file labeled 'Revised Manuscript with Track Changes'.

* An unmarked version of your revised paper without tracked changes. You should upload this as a separate file labeled 'Manuscript'.

If you would like to make changes to your financial disclosure, competing interests statement, or data availability statement, please make these updates within the submission form at the time of resubmission. Guidelines for resubmitting your figure files are available below the reviewer comments at the end of this letter.

We look forward to receiving your revised manuscript.

Kind regards,

Oliver Eales

Academic Editor

PLOS Computational Biology

Roger Kouyos

Section Editor

PLOS Computational Biology

If the reviewer comments include a recommendation to cite specific previously published works, please review and evaluate these publications to determine whether they are relevant and should be cited. There is no requirement to cite these works unless the editor has indicated otherwise.

**Response**: We thank the editor for the opportunity to revise our manuscript **entitled “***Heterogeneous suppressive effect of Wolbachia incompatible insect technique coupled with sterile insect technique across time and historical Ae. aegypti abundance - using distributional synthetic controls***” again.** **We hope that the revised manuscript below provides clarity and cogency to our study and adheres to the requirements of the journal.**

**Comment:** Please ensure that the CRediT author contributions listed for every co-author are completed accurately and in full.

At this stage, the following Authors/Authors require contributions: Yichen Zhai, Chia-Chen Chang, Jue Tao Lim, Lee Ching Ng, Cheong Huat Tan, and Zhiyong Xi. Please ensure that the full contributions of each author are acknowledged in the "Add/Edit/Remove Authors" section of our submission form.

The list of CRediT author contributions may be found here: <https://journals.plos.org/ploscompbiol/s/authorship#loc-author-contributions>

**Response:** We thank the editor for the comment. We have now ensured that the authorship contributions have been added to the submission form.

**Comment:** You have indicated that data is available from chang_chia-chen@nea.gov.sg. Please can we ask you to provide us with a general contact email address for the data requests, so readers can request access in perpetuity. If a general email is not available, please provide a link to a website where readers can obtain access to data.

**Response:** We thank the editor for the comment. We have now added a link to a website where readers can obtain access to data.

“Readers can also contact the National Environment Agency via <https://www.nea.gov.sg/corporate-functions/Contact-Us> for general data requests”

**Comment:** Some material included in your submission may be copyrighted. According to PLOS’s copyright policy, authors who use figures or other material (e.g., graphics, clipart, maps) from another author or copyright holder must demonstrate or obtain permission to publish this material under the Creative Commons Attribution 4.0 International (CC BY 4.0) License used by PLOS journals. Please closely review the details of PLOS’s copyright requirements here: PLOS Licenses and Copyright. If you need to request permissions from a copyright holder, you may use PLOS's Copyright Content Permission form.

**Response:** We thank the editor for the comment. See response below on the use of maps.

**Comment:** Please respond directly to this email and provide any known details concerning your material's license terms and permissions required for reuse, even if you have not yet obtained copyright permissions or are unsure of your material's copyright compatibility. Once you have responded and addressed all other outstanding technical requirements, you may resubmit your manuscript within Editorial Manager.

**Response:** We thank the editor for the comment. See response below on the use of maps.

**Comment:** Potential Copyright Issues:

i) Figures S11-S19: please (a) provide a direct link to the base layer of the map (i.e., the country or region border shape) and ensure this is also included in the figure legend; and (b) provide a link to the terms of use / license information for the base layer image or shapefile. We cannot publish proprietary or copyrighted maps (e.g. Google Maps, Mapquest) and the terms of use for your map base layer must be compatible with our CC BY 4.0 license.

Note: if you created the map in a software program like R or ArcGIS, please locate and indicate the source of the basemap shapefile onto which data has been plotted.

If your map was obtained from a copyrighted source please amend the figure so that the base map used is from an openly available source. Alternatively, please provide explicit written permission from the copyright holder granting you the right to publish the material under our CC BY 4.0 license.

If you are unsure whether you can use a map or not, please do reach out and we will be able to help you. The following websites are good examples of where you can source open access or public domain maps:

* U.S. Geological Survey (USGS) - All maps are in the public domain. (http://www.usgs.gov)

* PlaniGlobe - All maps are published under a Creative Commons license so please cite “PlaniGlobe, http://www.planiglobe.com, CC BY 2.0” in the image credit after the caption. (http://www.planiglobe.com/?lang=enl)

* Natural Earth - All maps are public domain. (http://www.naturalearthdata.com/about/terms-of-use/)

**Response: We thank the editor for the comment. We have now provided a direct link to the base layer of all maps presented in the manuscript and supplementary information. All maps were generated based on the base map provided by Urban Redevelopment Authority at the website (**<https://data.gov.sg/datasets?query=master+plan+2019+land+use+layer>**). This base map is following Open data licence and the licence information can be found at the website (**<https://data.gov.sg/open-data-licence>**).**

**Reviewer #1 Comments:**

The authors have done a thorough job addressing the concerns raised in the first round. The added sensitivity analyses (donor pool size, sampling distribution), the clarification of the IE measure and degenerate distribution, the bootstrap/permutation distinction, and the core-versus-buffer comparison are all satisfactory. I have only two minor remaining points before I can recommend acceptance.

**Comment:** Treatment assignment mechanism (previously Major Comment 4). The response letter clearly explains that Yishun and Tampines were selected for an expanding release strategy, while Bukit Batok and Choa Chu Kang were selected for targeted releases in areas with high *Ae. aegypti* abundance and persistent dengue transmission. However, this information does not appear in the manuscript itself. I ask the authors to add two to three sentences in the Methods section (around lines 97–119) describing the rationale for township selection and the different release strategies employed, citing Bansal et al. (Ref. 4) as appropriate. This context is important for readers who will not have access to the response letter.

**Response:** We thank the reviewer for the comment. We now added introduction of townships’ situations and the release strategies in the manuscript.

“*Data after EW26 2022, was excluded due to rollout of large-scale randomised controlled trials in the study setting in most high-risk locations in Singapore, which made construction of the synthetic control donor pool challenging^12,13^. In this field study, four townships were selected to test two different intervention release strategies sth– an expanding release strategy and a targeted release strategy. The expanding release strategy is implemented by progressively expanding the size of release areas over time whereas the targeted release strategy focused releases on areas with high mosquito abundance and persistent dengue transmission. Yishun and Tampines were two large towns selected for the expanding release strategy, whereas Bukit Batok and Choa Chu Kang were selected for the targeted release approach^4^. The spatial resolution used for analyses were traps within sectors, which comprise 10 – 20 public housing apartment blocks each and measure an average of approximately 0.088 km^2^.*”

**Comment:** Abstract inconsistency and residual typos (previously Minor Comment 5). The abstract (line 24) states that traps were “routinely maintained and measured on a fortnight basis,” but the revised Methods section (line 103) now states that each trap was “measured twice a week.” These are contradictory and the abstract must be corrected. Additionally, a few typos remain in the clean manuscript:

• Line 107: “Similar” → “Similarly”

• Line 242: “so that is can be scored” → “so that it can be scored”

• Line 242: “allowed us to calculating” → “allowed us to calculate”

I ask the authors to perform a careful final proofread before resubmission.

**Response:** We thank the reviewer for pointing out the grammatical errors. We have extensively checked through the manuscript and rectified grammatical errors where spotted. As these changes appeared throughout the manuscript, we have not quoted the changes herewith.

**Reviewer #3 Comments:**

**Comment:** My very minor comment is I think the abstract and author summary can ‘sell’ the developed method a bit better rather than only focus on how effective the treatment was – how this newly developed method differs to the previous one and what’s been added to the tools of intervention effectiveness evaluation available so far.

**Response:** We thank the reviewer for the comment. We have now provided more detail on the novelty of the statistical method used in the abstract and author summary:

“***Methods*** *Here, we developed a causal inference framework using distributional synthetic controls to explicitly account for spatio-temporal trap-level mosquito abundance data to ascertain the entomological efficacy of Wolbachia in suppressing Ae. aegypti abundance. This method is able to construct counterfactual distributions of intervened areas, provide detailed comparisons to actual distributions and quantify treatment effects of the intervention on mosquito abundance over different quantiles. By employing our framework to trap-level mosquito abundance data from 57,990 unique mosquito traps routinely maintained and measured twice a week, …*”

“***Conclusion*** *This study revealed spatial heterogeneities in suppressing wildtype female Ae. aegypti by IIT-SIT and provided strong evidence that IIT-SIT can drastically suppress wildtype Ae. aegypti populations despite heterogeneous treatment effects over time.*”

“***Author Summary*** *…We therefore developed a framework which can characterize differences in these intervention effects on a fine-spatial scale. This framework can provide new insights on the spatial heterogeneity of the treatment on mosquito abundance.*”

**Comment:** This part in SI is incomplete “However, we found similar trajectories in treatment effect. During the first 52-week treatment, average IE in core areas increased from 28.09% (95%CI: 17.78% – 40.81%) to 62.63% (95%CI: 48.41% – 81.78%), while average IE in buffer areas increased from 20.71% (95%CI: 11.52% – 29.65%).
“while average IE in buffer areas increased from 20.71% (95%CI: 11.52% – 29.65%)” from 20.71% to XXX”?

**Response**: We thank the reviewer for pointing out the missing number. We have now amended the text as follows:

*“During the first 52-week treatment, average IE in core areas increased from 28.09% (95%CI: 17.78% – 40.81%) to 62.63% (95%CI: 48.41% – 81.78%), while average IE in buffer areas increased from 20.71% (95%CI: 11.52% – 29.65%) to 80.00% (95%CI: 75.76% – 84.54%).”*
