# Supplementary material for: Heterogeneous suppressive effect of Wolbachia incompatible insect technique coupled with sterile insect technique across time and historical Ae. aegypti abundance - using distributional synthetic controls
Source: PLoS Comput Biol. 2026 Jun 22;22(6):e1014355. doi: 10.1371/journal.pcbi.1014355 (PMC13336471; doi:10.1371/journal.pcbi.1014355)
Supplement: Attachment — Submitted filename: Full_response_Apr27.docx [file pcbi.1014355.s007.docx]

**Editorial Letter**

**Comment:** Dear Dr Zhai,

Thank you for submitting your paper, "Heterogeneous suppressive effect of Wolbachia incompatible insect technique coupled with sterile insect technique across time and historical Ae. aegypti abundance - using distributional synthetic controls", to PLOS Computational Biology. We have checked over your submission and, before we can assign your manuscript to the Editor, we ask that you first please make the following modifications:

1) Please ensure that the affiliations of the authors listed on the manuscript title page (CITY) do exactly match with the affiliations provided in the online submission form

NOTE: Affiliations should include a department (if applicable), an institution, a CITY, and a country

2) Thank you for stating "The databases used in this study are the property of the National Environment Agency, Singapore. Permission to access mosquito abundance data should be obtained from the National Environment Agency, Singapore and interested parties may contact Chang Chia-Chen (chang_chia-chen@nea.gov.sg).

"

In the online submission form you indicate that your data is available from a co-author on this manuscript. According to our Data Policy, data must either be included with the submission, be placed in a public repository, or available from a third party contact point who must not be an author on the manuscript. Please revise your data statement to a non-author institutional point of contact, such as a data access or ethics committee, and send this to us via return email. Please also include contact information for the third party organization (this contact should be a central address and not a named person), and please include the full citation of where the data can be found.

3) Thank you for stating "The base layer of the maps in this study contains information from the Master Plan 2019 Land Use Layer dataset (https://data.gov.sg/datasets?query=master+plan+2019+land+use+layer) accessed on 25 April 2025, from the Urban Redevelopment Authority, which is made available under the terms of the Singapore Open Data Licence version 1.0 (https://data.gov.sg/open-data-licence)."

Please ensure the source details are added in the respective figure legends

Once you have made the necessary changes in Editorial Manager, please re-approve the submission by following the same steps as before.

Please resolve these issues and resubmit your manuscript within 30 days. If you need more time, please email the journal office at ploscompbiol@plos.org. We are happy to grant extensions of up to one month past this due date. If we do not hear from you within 30 days, we will withdraw your manuscript on your behalf.

If you have any questions or require assistance, please don't hesitate to get in touch. To help us minimize inbox volumes, we kindly ask that you not reply to this email simply to confirm you have resubmitted your revisions.

Kind regards,

Nithya Venkatesan

PLOS Computational Biology

[ploscompbiol@plos.org](mailto:ploscompbiol@plos.org)

**Response**: We thank the editor for the opportunity to revise our manuscript **entitled “***Heterogeneous suppressive effect of Wolbachia incompatible insect technique coupled with sterile insect technique across time and historical Ae. aegypti abundance - using distributional synthetic controls***” again. We hope that the revised manuscript below provides clarity and cogency to our study and adheres to the requirements of the journal.**

**We thank the editor for the reminder. We apologize for omitting the term “city” and have added the city information for the affiliations in the submission form.**

**We thank the editor for the comment on the data availability statement. The statement has already provided one non-author institutional point of contact.**

**“… *Readers can also contact the National Environment Agency via https://www.nea.gov.sg/corporate-functions/Contact-Us for general data requests.*”**

**We thank the editor for the comment about the map copyright issue. We added copyright statements to the figure legends with the maps accordingly.**

**Figure 3, SI Figure 11 – 19 *“… Base layer from the Master Plan 2019 Land Use Layer dataset, licensed under the Singapore Open Data Licence version 1.0.”***
