# Supplementary material for: Heterogeneous suppressive effect of Wolbachia incompatible insect technique coupled with sterile insect technique across time and historical Ae. aegypti abundance - using distributional synthetic controls
Source: PLoS Comput Biol. 2026 Jun 22;22(6):e1014355. doi: 10.1371/journal.pcbi.1014355 (PMC13336471; doi:10.1371/journal.pcbi.1014355)
Supplement: Attachment — Submitted filename: Full_response_May13.docx [file pcbi.1014355.s008.docx]

**Comments:** PCOMPBIOL-D-25-02450R3
Heterogeneous suppressive effect of Wolbachia incompatible insect technique coupled with sterile insect technique across time and historical Ae. aegypti abundance - using distributional synthetic controls
PLOS Computational Biology

Dear Dr. Zhai,

Thank you for submitting your manuscript to PLOS Computational Biology. After careful consideration, we feel that it has merit but does not fully meet PLOS Computational Biology's publication criteria as it currently stands. Therefore, we invite you to submit a revised version of the manuscript that addresses the following points that I noted in my previous decision letter, but were not addressed in the revisions. These have been provided below.

Please submit your revised manuscript by Jul 05 2026 11:59PM. If you will need more time than this to complete your revisions, please reply to this message or contact the journal office at [ploscompbiol@plos.org](mailto:ploscompbiol@plos.org). When you're ready to submit your revision, log on to <https://www.editorialmanager.com/pcompbiol/> and select the 'Submissions Needing Revision' folder to locate your manuscript file.

Please include the following items when submitting your revised manuscript:

* A letter that responds to each point raised by the editor and reviewer(s). You should upload this letter as a separate file labeled 'Response to Reviewers'. This file does not need to include responses to formatting updates and technical items listed in the 'Journal Requirements' section below.
* A marked-up copy of your manuscript that highlights changes made to the original version. You should upload this as a separate file labeled 'Revised Manuscript with Track Changes'.
* An unmarked version of your revised paper without tracked changes. You should upload this as a separate file labeled 'Manuscript'.

If you would like to make changes to your financial disclosure, competing interests statement, or data availability statement, please make these updates within the submission form at the time of resubmission. Guidelines for resubmitting your figure files are available below the reviewer comments at the end of this letter.

We look forward to receiving your revised manuscript.

Kind regards,

Oliver Eales
Academic Editor
PLOS Computational Biology

Roger Kouyos
Section Editor
PLOS Computational Biology

**Response**: We thank the editor for the opportunity to revise our manuscript **entitled “***Heterogeneous suppressive effect of Wolbachia incompatible insect technique coupled with sterile insect technique across time and historical Ae. aegypti abundance - using distributional synthetic controls***”. We hope that the revised manuscript below provides clarity and cogency to our study and adheres to the requirements of the journal.**

**Additional Editor Comments 1**: "There are some minor revisions required for the figures and figure legends before the article can be accepted. Currently the figure captions do not provide enough detail to interpret the figures. For example Figure 2 and 4 have confidence intervals (I assume) around each line as a shaded region, but the caption and figure does not state what the shaded region is, or what confidence level it represents (e.g. 95% confidence intervals?). Figure 5 has box and whiskers, but their meaning has not been explained in the caption.

**Response:** We thank the editor for the comment. We have now explained in the caption and figures what the shaded regions are for Figure 2, 4 **as shaded and bolded below**:

***Figure 2***

(A) *IIT-SIT intervention effectiveness to reduce Ae. aegypti abundance by time-since-intervention in directly intervened sectors by GAI quantile*
(B) *IIT-SIT intervention effectiveness to reduce Ae. aegypti abundance by time-since-intervention in spillover sectors by GAI quantile*
(C) *Raw reduction distribution in Ae. aegypti abundance by time-since-intervention in directly intervened sectors*
(D) *Raw reduction distribution in Ae. aegypti abundance by time-since-intervention in spillover intervened sectors*

*The coloured lines represent the average values across the sectors.* ***The shaded regions represent the pointwise 95% confidence intervals. The shaded regions in grey represent quantiles where there is an insignificant reduction in Ae. aegypti populations due to intervention. The shaded regions in other colours represent quantiles where there is a significant reduction in Ae. aegypti populations due to intervention.***

*Plots (A), (B) have been truncated as no intervention effectiveness estimates are negative and have 95% CIs which are not bounded by 0.*

***Figure 4***

*(****A****) Sector intervention effectiveness of IIT-SIT to reduce Ae. aegypti abundance over previous sector relative Ae. aegypti abundance quantile in direct intervened sectors. Coloured lines represent GAM models based on corresponding post-intervention IE. Each dot represents a unique sector evaluated at that specific event-time.*

*(****B****) Mean intervention effectiveness of IIT-SIT to reduce in Ae. aegypti abundance in direct intervened sectors by township.* ***Coloured lines represent average IE across the sectors in each township. The shaded areas represent pointwise 95% confidence intervals.***

***Figure 5***

*(****A****) Increase in the percentage of traps recording no mosquitoes by time-since-intervention in directly intervened sectors*

*(****B****) Increase in the percentage of traps recording no mosquitoes by study time in directly intervened sectors*

*(****C****) Increase in the percentage of traps recording no mosquitoes by time-since-intervention in sectors located adjacent to directly intervened sectors*

*(****D****) Increase in the percentage of traps recording no mosquitoes by study time in sectors located adjacent to directly intervened sectors*

*Outliers are not shown in the graphs.* ***Lines in the boxes represent the middle 50% of the estimate, whereas the ends of the boxes represent the interquartile range, spanning from the 25th percentile to the 75th percentile. The lines represent the minimum and maximum respectively.***

**Additional Editor Comments 2:** Figure 2B has the shaded region missing on some of the lines? Figure 2D has a different colour shaded region around the bottom most line compared to the other panels (and the legend)."

**Response: We thank the editor for the comments.** Figure 2A and B has the bottom portion of the 1–13 weeks intervention effectiveness estimates truncated in the graph as they are not significant and contribute to visual noise in the figure. We have now stated this in the caption. Furthermore, the grey portion in Figure 2D has been explained in the caption previously, **as shaded and bolded below**:

***Figure 2***

(A) *IIT-SIT intervention effectiveness to reduce Ae. aegypti abundance by time-since-intervention in directly intervened sectors by GAI quantile*
(B) *IIT-SIT intervention effectiveness to reduce Ae. aegypti abundance by time-since-intervention in spillover sectors by GAI quantile*
(C) *Raw reduction distribution in Ae. aegypti abundance by time-since-intervention in directly intervened sectors*
(D) *Raw reduction distribution in Ae. aegypti abundance by time-since-intervention in spillover intervened sectors*

*The coloured lines represent the average values across the sectors. The shaded regions represent the pointwise 95% confidence intervals. The shaded regions in grey represent quantiles where there is an insignificant reduction in Ae. aegypti populations due to intervention. The shaded regions in other colours represent quantiles where there is a significant reduction in Ae. aegypti populations due to intervention.*

***Plots (A), (B) have been truncated as the intervention effectiveness estimates are negative and have 95% CIs which are not bounded by 0.***
